# Mechanisms responsible for the ability of enoxaparin sodium to inhibit inflammatory responses in the immune microenvironment of bone repair: A transcriptomic sequencing study

**DOI:** 10.1371/journal.pone.0332041

**Published:** 2025-09-08

**Authors:** Xiaoyu Shen, Qiang Yao, Lijie Ma

**Affiliations:** 1 Orthopaedics, Hebei Medical University Third Hospital, Shijiazhuang, China; 2 Internal Medicine, The First Hospital of Hebei Medical University, Shijiazhuang, China; Southern Medical University Nanfang Hospital, CHINA

## Abstract

Enoxaparin sodium (ES), a low molecular weight heparin derivative, has recently been recognized for its diverse biological activities. In particular, the ability of heparin to modulate inflammation has been utilized to enhance the biocompatibility of bone implant materials. In this study, we utilized poly (methyl methacrylate) (PMMA), a drug loading bone implant material, as a matrix and combined this with enoxaparin sodium (ES) to create enoxaparin sodium PMMA cement (ES-PMMA) to investigate the regulatory effects of ES on inflammatory responses in bone tissue from an animal model. We established a rabbit model of femoral condyle bone defects to investigate the immunoregulatory mechanisms of ES-PMMA. Rabbits were divided into control (n = 5), model (n = 10), PMMA (n = 10) and ES-PMMA (n = 10) groups. The control group underwent sham surgery as a blank control, while the model group was established with a bone defect model in the rabbit femoral condyle. The PMMA group and ES-PMMA group followed the same modeling procedure as the model group. After successful modeling, the PMMA group and ES-PMMA group were implanted with PMMA bone cement columns and ES-PMMA bone cement columns, respectively. Ten days post-surgery, cancellous bone tissue from the defect site was collected from each group, and the control group was sampled at the same location. Tissue samples were collected from each group for transcriptomic sequencing. RNA sequencing (RNA-seq) was performed and differentially expressed mRNAs were identified between the model and controls, between the PMMA and model groups, and between the ES-PMMA and model groups. Key candidate genes associated with ES-PMMA treatment were identified (304 genes), and Gene Set Variation Analysis (GSVA), Gene Ontology (GO), and Kyoto Encyclopedia of Genes and Genomes (KEGG) pathway enrichment analyses were performed on the differentially expressed genes and key candidate genes in each group (P < 0.05). The 304 key candidate genes associated with ES-PMMA treatment are involved in functions such as inflammation, cell proliferation, and differentiation. Protein-protein interaction (PPI) network analysis and machine learning revealed three key candidate genes in the ES-PMMA group: recombination activating gene (*RAG1*), Src-like adaptor 2 (*SLA2*), S100 calcium binding protein and beta (neural) (*S100B*). SLA2 and RAG1 are known to be related to inflammation, whereas S100B is related to osteogenic differentiation. Finally, the subcellular localization and functional similarities of the three genes were assessed, and their transcription factors and miRNAs were predicted. Collectively, these findings provide insights into the mechanism of ES in regulating immune responses in the bone; this may facilitate the development of novel bone implant materials.

## 1. Introduction

Osteoimmunology is an emerging interdisciplinary field that aims to utilize the important relationship between signaling molecules in the bone and the immune system. In the bone marrow ‘niche’, defined as the specialized microenvironment in which osteocytes, immune cells, and hematopoietic stem cells interact to regulate bone homeostasis and immune responses, osteocytes and immune cells share the same microenvironment, interact with each other, and cooperate to perform specific functions [1,2]. Bone cells and immune cells are known to regulate each other. For example, mechanical stimulation can promote lymphopoiesis by stimulating osteogenic progenitor cells [3]. The immune system is involved in all stages of bone wound repair. In the early and middle stages of bone repair, M1 macrophages promote the recruitment, migration, and early differentiation of bone mesenchymal stem cells (BMSCs). In the later stage of bone repair, M2 macrophages play a key role in the osteogenic differentiation of BMSCs [4,5]. However, the excessive activation of inflammatory responses in the bone immune system can lead to a multitude of orthopedic diseases, including rheumatoid arthritis, the activation of osteoclasts by inflammatory mediators, and bone resorption, thus resulting in inflammatory osteoporosis, bone destruction and other issues [6].

Owing to an increase in the incidence of bone diseases caused by congenital malformations, bone tumors, arthritis and bone injuries resulting from road accidents, more than four million bone graft or bone replacement surgeries are performed each year globally, making the demand for bone implant materials extremely high [7]. Bone implant materials, as foreign substances, are often limited by their poor tissue compatibility. This incompatibility frequently triggers inflammatory responses, which in turn leads to a series of complications, including inflammatory osteoporosis and bone destruction, ultimately resulting in implant failures, such as the loosening of joint prostheses. Poly (methyl methacrylate) (PMMA) is a common bone implant material in orthopedic surgery, and is widely used in artificial joint replacement, spine surgery and trauma repair. As a foreign body, PMMA has poor biocompatibility, activates inflammatory responses, and induces toxicity owing to residual monomers, eventually often leading to the excessive activation of inflammatory responses and implant rejection [8–12]. However, PMMA also possesses numerous advantages, including a porous structure, stability, and exceptional drug-loading and release properties kinetics, making it an ideal candidate for modification and application as a matrix in bone implant materials [13]. Furthermore, its capacity as a drug-carrying material allows for the controlled release of therapeutic agents directly to the surrounding bone tissue [14]. Consequently, PMMA is frequently employed as a matrix to develop novel bone implant materials and serves as a benchmark for evaluating the advantages and disadvantages of new bone implant materials, given that PMMA possesses exceptional drug-loading efficiency (up to 20% w/w) and sustained release kinetics (60–80% drug release over 7–14 days) [13–15].

Heparan sulfate (HS) is widely expressed on cell surfaces and in the extracellular matrix of cells [16]. Due to its structural diversity and conformational flexibility, HS and its derivatives, such as heparin (Hep), can participate in multiple signaling pathways, and some of its derivatives have been used as additives in bone implants to enhance the bioactivity of implant materials in bone tissue [16,17]. The molecular structure of HS includes numerous sulfate and carboxyl groups, and its surface exhibits a highly negative charge. HS does not only adsorb many important biomolecules via a specific binding domain; it can also bind to the heparin-binding domains of several proteins, including thrombin, lipoprotein, growth factors, chemokines, viral coat proteins and proteins of the extracellular matrix [18–20]. Previous studies have shown that its derivatives can promote wound healing and enhance the effects of growth factors [21]. HS and its derivatives have been proven to regulate the differentiation, proliferation, migration and apoptosis of T Lymphocytes (T) in a manner that is independent of anticoagulant activity [22]. Low molecular weight heparin (LMWH) can modulate immune responses by increasing pro-inflammatory Th1-associated chemokines, potentially influencing T-cell polarization during inflammatory conditions. Interleukin-4 (IL-4), IL-5, and IL-13 are all cytokines secreted by Th2 cells that can inhibit the production of osteoclasts [23,24]. Furthermore, HS can regulate bone homeostasis by interacting with adhesion-related proteins in osteoprogenitor cells to modulate their ability to undergo osteogenic differentiation [25]. Furthermore, HS can bind to fibroblast growth factor 2 (FGF-2) and the fibroblast growth factor receptor (FGFR) in osteoblasts to activate extracellular regulated protein kinases1/2 (ERK1/2) signaling pathway downstream, thereby enhancing the proliferation and function of osteoblasts and promoting the growth and regeneration of bone tissue [26]. Other research has demonstrated that heparin is a strong inhibitor of osteoclast formation by maintaining active ERK signaling [27–29]. When used locally in bone defects, HS can protect endogenous growth factors from being degraded by proteases and promote osteogenic differentiation, thereby facilitating bone repair and regeneration [30]. Collectively, these data imply that HS and its derivatives plays an important role in regulating bone immunity. In clinical practice, HS has already been used as an additive in bone implant materials and other applications [26].

Enoxaparin sodium (ES), a low molecular weight heparin (4–6 kDa) with reduced chain heterogeneity, is a form of heparan sulfate (HS) that exhibits enhanced biological activity compared to conventional heparin due to its abundance of sulfate groups and unique molecular conformation [31,32]. As a widely utilized anticoagulant in clinical settings, the specific impact of enoxaparin sodium (ES) on bone tissue remains a subject of controversy. While some researchers have found that long-term use of ES may be associated with osteoporosis, others have discovered that ES has osteogenic induction effects on BMSCs [33,34]. In our previous research, we investigated the effect of ES on inflammatory responses in bone tissue and showed that a combination of ES and PMMA (ES-PMMA) yielded good ES-release ability, could maintain a clinically relevant drug concentration locally over a certain time period, and had similar mechanical strength to traditional PMMA [35,36]. Preliminary findings from our previous research showed that ES-PMMA exerted local anti-inflammatory effects by releasing ES, which inhibited the production of inflammatory factors (IL-6 and IL-1) and tumor necrosis factor-α (TNF-α) [32,36,37]. Investigating the anti-inflammatory role of ES in bone implants, such as PMMA, is vital because inflammation in surrounding tissues triggered by bone implant materials poses a major challenge and can lead to implant failure. ES can potentially enhance bioactivity by inhibiting excessive inflammation [38].

In the present study, we aimed to validate ES as an innovative implant additive by identifying mechanisms that could specifically reduce PMMA-induced inflammation; this approach would benefit from the cost-effectiveness and anticoagulant properties of ES. Furthermore, we aimed to elucidate the inflammatory mechanisms triggered by bone implants during repair, thus improving our understanding of osteoimmunity. Due to the need for a sufficient volume of ES-PMMA to ensure the prolonged release of ES and adequate bone tissue sampling, we selected rabbits as the experimental animal model for our research. In addition, we used gene sequencing to identify potential biomarkers and signaling pathways related to the anti-inflammatory effects of ES. Clinically, the application of ES as an additive could lead to the development of advanced bone implants with better biocompatibility and lower risks of inflammation. Overall, our findings contribute to the broader field of biomaterial science by highlighting the potential of heparin-like compounds in developing novel bioactive bone implants.

## 2. Materials and methods

### 2.1. Preparation of the rabbit model and RNA sample collection

**Fig 1:**
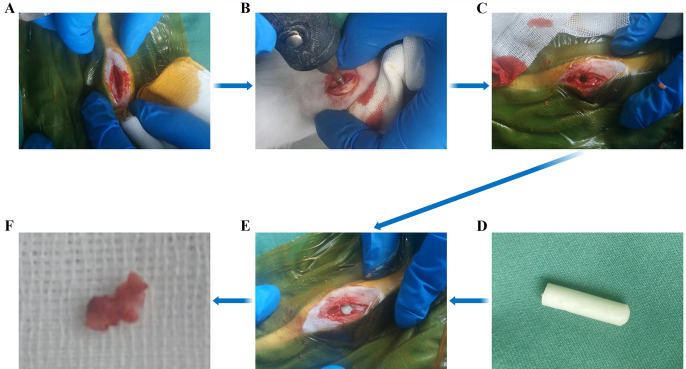
Surgical procedure and tissue sample collection in rabbits. A. A lateral incision was made in the distal femur of the rabbit, and the skin and subcutaneous tissues were dissected layer by layer to fully expose the bone tissue. B. A drill was used to create a bone defect by drilling perpendicular to the bone surface. C. The bone defect was refined to achieve a diameter of 3 mm and a depth of 1 cm. D. According to the ratio established in our previous study (monomer-to-polymer ratio of 2:1), we prepared Polymethylmethacrylate (PMMA)/Enoxaparin Sodium-Polymethylmethacrylate (ES-PMMA) cylindrical implants with a diameter of 3 mm and a length of 1 cm. E. The cylindrical implant was inserted into the bone defect, and the subcutaneous tissues and skin were sutured layer-by-layer, followed by wound dressing. F. After allowing for a period of 10 days for healing and integration, we harvested cancellous bone tissue around the molded area in the distal femur.

New Zealand rabbits, aged 6–7 months, weighing 2.5–3 kg, and exhibiting normal limb movement (assessed via visual observation, gait analysis, and functional tests), were selected for all in vivo experiments. The animals were sourced from Wangdu Tong Hui Animal Breeding Co. Ltd. (Baoding City, China; Animal Certificate number: 210509). The animal experimental protocol was approved by the Medical Ethics Committee of the Third Hospital of Hebei Medical University (Approval number: z2023-012–01). All experimental procedures involving animals were conducted in accordance with the ethical standards set forth by the Animal Experimentation Ethics Committee of the Third Hospital of Hebei Medical University, Shijiazhuang, China (reference number: z2023-012-1). The rabbits were maintained in standard laboratory conditions with a controlled temperature at 18–22°C, humidity at 50–60%, and a 12-hour light/dark cycle. The rabbits were fed a standard laboratory rabbit diet provided by Qingdao Kangda Aibo Biotechnology Co., Ltd. (Qingdao, China; License No.: SCXK(lu)20220004) and had access to water *ad libitum*. Rabbits were randomly allocated to four groups using computer-generated randomization (R v4.3.0; seed = 2024), which were sorted to assign animals to the Control, Model, PMMA, and ES-PMMA groups, ensuring unbiased distribution [38]: a blank control group (n = 5), a model group (n = 10), PMMA (n = 10), and ES-PMMA (n = 10) groups. For anesthesia and analgesia, animals were anesthetized using urethane (Shanghai Yuanye Bio-Technology Co., Ltd., Shanghai, China; Catalog No.: S30146) administered via marginal ear vein injection at a dose of 1.5 g/kg body weight. Postoperative pain was managed by the local injection of lidocaine at the surgical site. A blank group served as the control group, for which only the skin and subcutaneous tissue of the distal femur were incised without damaging the bone. Subsequently the wound was meticulously sutured layer-by-layer, and antibiotics were administered for 24 hours to prevent infection. Samples were obtained directly from the cancellous bone tissue of the distal femur. In the model group, a bone defect was created at the distal femur to simulate bone injury. A circular bone defect, with a diameter of 3 mm and a depth of 1 cm, was carefully formed by sequentially removing layers of skin, subcutaneous tissue, and periosteum at the lateral end of the femur. The incisions were then closed in layers. To minimize animal suffering, the following measures were implemented: (1) preoperative fasting for 6 hours to reduce stress during anesthesia; (2) daily monitoring of wound healing, mobility, and behavior post-surgery; and (3) prompt euthanasia upon completion of the study to avoid prolonged distress. After 10 days, and in accordance with the guidelines set by the Institutional Animal Care and Use Committee (IACUC), the rabbits were euthanized humanely via the intravenous injection of KCL (10% solution) following the completion of tissue collection. Cancellous bone tissue surrounding the bone defect in the distal femur was collected for analysis. Rabbits in the PMMA group were treated in a manner similar to the model group, with the exception that a PMMA mold was implanted into the bone defect to simulate its application for treating such defects. A cylindrical PMMA mold, with a diameter of 3 mm and a length of 1 cm, was inserted into the bone defect at the distal femur. Following a period of 10 days for healing and integration, the cancellous bone tissue surrounding the mold in the distal femur was harvested. For the ES-PMMA Group, we inserted an ES-PMMA implant into an artificially created bone defect to mimic its application to treat these defects. ES-PMMA was prepared as described previously [26–28]. In brief, enoxaparin sodium (ES) was incorporated into poly (methyl methacrylate) (PMMA) at a concentration of 5% w/w under controlled polymerization conditions (monomer-to-polymer ratio of 2:1, polymerization temperature of 37°C). The mixture was molded into cylindrical implants (3 mm diameter × 1 cm length) to ensure consistent drug release and mechanical strength. A cylindrical ES-PMMA implant (3 mm diameter × 1 cm length) was placed inside the manufactured bony lesion located at the distal femur. Ten days post-surgery, the cancellous bone tissue surrounding the molded region in the distal femur was harvested for analysis. All samples were placed in Trizol Reagent (Invitrogen, Carlsbad, CA, USA) and sent to Beijing Zhongke Shengxin Biotechnology Co. Ltd. for sequencing as shown in Fig 1, which depicts the steps from incision to implant insertion and sample harvesting.

### 2.2. Quality control procedure for RNA sequencing

Total RNA was extracted using TRIzol reagent (Invitrogen), and RNA integrity was evaluated using an Agilent 2100 Bioanalyzer (Agilent Technologies). Only samples with an RNA Integrity Number (RIN) ≥ 7.0 were included for library construction. Ribosomal RNA contamination was assessed by aligning raw reads to the SILVA rRNA database, and samples with > 5% rRNA reads were subjected to ribosomal depletion. Raw sequencing data were pre-processed using Trimmomatic software (v0.39) (http://www.usadellab.org/cms/?page=trimmomatic) with default parameters to remove splice contaminants and low-quality reads. Filtered clean reads were then aligned to the human reference genome (GRCh37) using Hisat2 software (v2.2.1) (https://daehwankimlab.github.io/hisat2/), with the following key parameters: --outFilterMismatchNmax 2 (maximum 2 mismatches per read), --alignIntronMin 20 (minimum intron size 20 bp), --alignIntronMax 1000000 (maximum intron size 1 Mb), and --outFilterMultimapNmax 10 (allow up to 10 multi-mapping locations per read). The alignment between each sample and the reference genome were compiled into a table for comparative analysis. Reads with a mapping quality (MAPQ) < 30 were filtered out. Strand-specificity was confirmed using RSeQC. Subsequently, gene expression profiles were extracted using Feature Count software (v2.0.0) (https://sourceforge.net/projects/subread/), with default parameters.

### 2.3. Identification of differentially expressed genes (DEGs)

To model and identify sequence differences between the normal, model, PMMA, and ES-PMMA groups, principal component analysis (PCA) in R package (https://cran.r-project.org/) was first performed for data visualization and outlier detection, followed by DEG analysis. The screening thresholds for DEGs were as follows: (1) statistical significance (p < 0.05), (2) fold change (|log2FC| > 1), (3) adjusted p-value (Benjamini-Hochberg correction for multiple testing), (4) replication number (biological replicates ≥3 for reliability), (5) normalization (TMM/FPKM for library size correction), and (6) filtering (low-expression gene removal).

### 2.4. Differential expression analysis and the identification of key candidate genes associated with ES-PMMA

The DESeq2 package in R software was used to identify DEGs between the model and control groups, between the PMMA and model groups, and between the ES-PMMA and model groups, based on transcriptomic sequencing data. These gene sets were named DEG-1, DEG-2, and DEG-3, respectively. The screening threshold for DEGs was set to P < 0.05 and a |log2FC| > 0.5. A volcano plot and a heat map were then generated in the DESeq2 package in R to visualize the DEGs. We also generated a Venn diagram to identify the intersection between DEG-1 and DEG-2 to identify genes specifically associated with traditional PMMA. Subsequently, another Venn diagram was generated to identify the intersection between DEG-1 and DEG-3 to identify genes specifically associated with ES-PMMA. Finally, key candidate genes associated with ES-PMMA were obtained by excluding common genes between genes specifically associated with ES-PMMA and genes specifically associated with PMMA from the ES-PMMA gene set.

### 2.5. Identification of candidate genes by protein-protein interaction (PPI) network analysis and correlation analysis

To investigate the interaction between key candidate genes associated with ES-PMMA, we next constructed a PPI network using the STRING database (https://string-db.org/). STRING-derived interaction data were imported into Cytoscape (https://cytoscape.org/), in which the Degree, Closeness, and Betweenness algorithms were used to score the genes. “Degree” represents the number of proteins that gene nodes directly interact with in the PPI network. “Closeness” is used to measure the average length of the shortest path from this gene to all other genes in the network. “Betweenness” is used to measure the frequency of gene occurrence in all the shortest paths, which indicates the key regulatory factor. Three centrality algorithms quantify the influence of genes in the PPI network from different dimensions. The combined use can comprehensively identify the key regulatory factors under the background of differential expression. The top 20 genes identified by each algorithm were intersected, thus resulting in the identification of 12 candidate genes. Subsequently, Spearman’s correlation analysis was performed to investigate the relationship between candidate genes in the ES-PMMA–model dataset.

### 2.6. Identification of key genes by machine learning

Three machine learning algorithms were used to identify key genes from the candidate gene set in the ES-PMMA dataset: LASSO regression, support vector machine recursive feature elimination (SVM-RFE), and Boruta. The analysis object in the machine learning stage was the gene expression data of the model group (Model, n = 10) and the ES-PMMA group (Heparin, n = 10). No independent training set and test set division was conducted; instead, 10-fold cross-validation was used to evaluate the stability of the model. Ten-fold cross-validation was implemented during model training to optimize hyper parameters and prevent overfitting. Model performance was quantified using the Area Under the Receiver Operating Characteristic Curve (AUC-ROC) and precision-recall curves. SVM-RFE is a recursive feature elimination algorithm based on Support Vector Machine (SVM) that removes features with the lowest contribution iteratively, gradually selecting the most critical genes for classification (between the model group and the ES-PMMA group). Our analysis involved a linear kernel and a feature elimination step size set so that 10% of the lowest-ranked features were removed in each iteration, thereby gradually reducing the feature set to enhance the generalization ability of the model. Boruta was used to identify potential key genes most closely related to the therapeutic effects of ES-PMMA. The number of iterations was 1000 and Z-score (importance score) was calculated for each real gene and shadow feature. When the feature Z value was> than the maximum shadow feature Z value and p was < 0.01, this condition was marked as “confirmed” (a significant important feature). Next, the glmnet package in R was used to implement LASSO analysis. The optimal λ that minimized the error of the validation set was selected by 10-fold cross-validation. The regularization path comprised 100 exponentially decreasing λ values with an elastic net parameter α of 1 (pure LASSO). Features with non-zero coefficients (|β| > 0) were retained. Finally, the genes identified by the three machine learning algorithms were intersected.

### 2.7. ROC analysis, nomogram construction, and expression analysis of biomarkers

The pROC package in RStudio (https://www.rstudio.com/) was used to generate ROC curves to evaluate the ability of key genes to distinguish between the ES-PMMA group and the model group; then, the area under the ROC curve (AUC) was calculated with 95% confidence intervals. An AUC value > 0.7 was considered to indicate that a given key gene had good discrimination ability. Additionally, precision-recall curves (PRC) were generated using the PRROC package (https://cran.r-project.org/web/packages/PRROC/) to assess performance under class imbalance. The area under the precision-recall curve (AUPRC) was computed, with AUPRC values > 0.5 (above random chance) considered clinically meaningful. For the selected key genes, their ability to distinguish between the model group and the ES-PMMA group was evaluated. The dataset used was the same as that in the machine learning stage. The nomogram was constructed based on these key genes, and a risk prediction model was built in the same dataset (the model group and the ES-PMMA group), without introducing a new dataset division. The Hosmer-Lemeshow test was used for calibration metrics. Finally, we constructed a transcription factor (TF)-biomarker-microRNA (miRNA) regulatory network. We used this network to investigate whether a given biomarker was regulated by a TF and identify the interaction between biomarkers and miRNA, based on the GhEA3 predictive biomarkers of TFs (https://maayanlab.cloud/chea3/) database.

### 2.8. RNA isolation and real-time quantitative PCR (RT-qPCR)

To further verify the expression levels of the key candidate genes associated with ES-PMMA, an additional cohort of animals (n = 6 per group) was subjected to the same surgical and sampling procedures as previously described in Section 2.1. All additional procedures were approved by the Third Hospital of Hebei Medical University (Approval number: z2023-012–01) and complied with the ARRIVE guidelines. Bone tissues were collected, and total RNA was extracted using TRIzol reagent (Invitrogen, USA) in accordance with the manufacturer’s protocol. RNA concentration and purity were assessed spectrophotometrically (NanoDrop, Thermo Fisher Scientific). cDNA was synthesized from 1 µg of total RNA using a SureScript First-Strand cDNA Synthesis Kit (GeneCopoeia, USA) in accordance with the manufacturer’s instructions.

RT-qPCR was performed to determine the expression levels of *SLA2*, *RAG1* and *S100B*, using a Bio-Rad iQ5 Real-Time PCR system (Bio-Rad, USA) with 2 × SYBR Green qPCR MasterMix (Applied Biosystems, USA). The reaction mixture (20 µL) contained 10 µL MasterMix, 1 µL each of forward and reverse primers (10 µM), 2 µL of cDNA template, and 6 µL of nuclease-free water. The thermal cycling conditions were as follows: 95°C for 10 min (initial denaturation), followed by 40 cycles of 95°C for 15 sec, 55°C for 30 sec, and 72°C for 30 sec. *GAPDH* was used as a reference gene for normalization. All reactions were performed in triplicate, and no-template controls were included. Primer sequences for target genes (*SLA2*, *RAG1*, *S100B*) and *GAPDH* were designed and synthesized by Wuhan Jinkaruo Biological Engineering Co., Ltd (Table 1). Relative mRNA expression levels were calculated using the 2 − ΔΔCT method [36].

**Table 1:** Primer sequences for target genes in rabbit (Oryctolagus cuniculus).

Gene	Forward Primer (5′ → 3′)	Reverse Primer (5′ → 3′)	Amplicon (bp)	Tm (°C)	ACCESSION
*GAPDH*	GGCACAGTCAAGGCTGAGAATG	ATGGTGGTGAAGACGCCAGTA	150	58–62	NM_001082253.1
*S100B*	AAGTCCACACCCAGTCCTCT	GTGCTTGTCACCCTCTCTCC	198	58–62	NM_013191.2
*RAG1*	GGAAGACATCTTGGACGGCA	CCCGTGCTTCTCACTCACAT	118	58–62	NM_053468.2
*SLA2*	GTTGAAGTTTGGTTCAGTGTGC	GCTTGGGCTGGAGTTTTTCC	87	57–60	XM_063284392.1

### 2.9. Statistical analysis

Statistical analysis was conducted using SPSS 26.0 (IBM Corp., Armonk, NY, USA). One-way ANOVA was used to conduct an overall difference test on multiple groups (including the Control, Model, PMMA, and ES-PMMA groups), and then pairwise comparisons between groups were performed using Tukey’s post-hoc test (such as a direct comparison between the ES-PMMA group and the Model group). To control for the false positives caused by multiple tests, the FDR (False Discovery Rate) correction (i.e., the Benjamini-Hochberg correction) was adopted to ensure the reliability of the statistical results in the paper. A corrected p-value < 0.05 was set as the significance threshold. For RT-qPCR analysis, all quantitative PCR data were normalized to the reference genes *GAPDH* using the 2^(-ΔΔCt) method. Statistical analyses were performed using one-way ANOVA followed by Tukey’s multiple comparisons test for multiple comparisons after confirming normality (Shapiro-Wilk test, all p > 0.05) and homogeneity of variance (Levene’s test, all p > 0.1). All analysis were conducted using GraphPad Prism 9.0 with statistical significance set at α = 0.05 (two-tailed).

## 3. Results

### 3.1. Quality control of sequencing data

**Fig 2:**
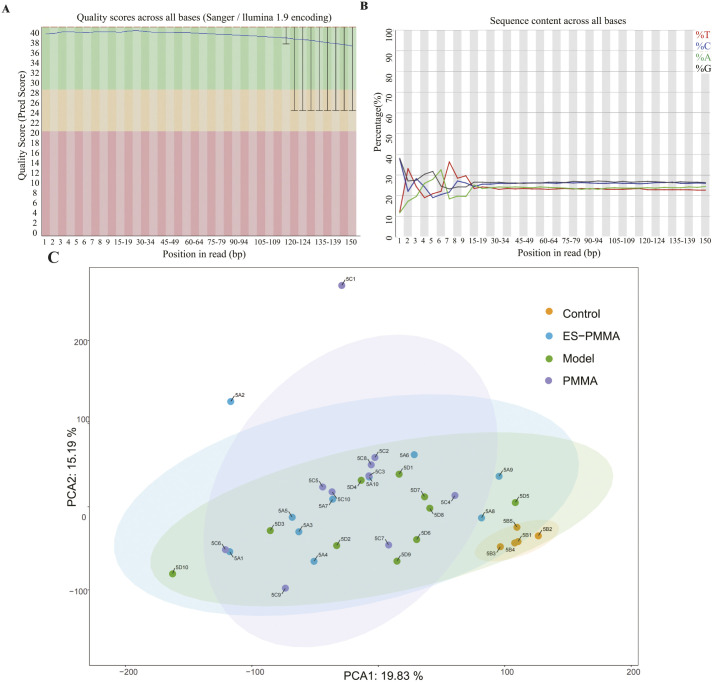
Quality control. (A) Distribution of quality scores for base positions. The x-axis represents the relative position of each base in the reads, whereas the y-axis represents the sequencing quality scores (higher scores indicate better base quality, with a 1% error rate at a quality score of 20 and a 0.1% error rate at a quality score of 30); (B) Nucleotide composition plot showing the position of reads on the x-axis and the proportion of single bases on the y-axis. Different colors indicate various types of bases; (C) Principal Component Analysis (PCA) distribution map; yellow, blue, green, and purple indicate the Polymethylmethacrylate (PMMA), control, Enoxaparin Sodium-Polymethylmethacrylate (ES-PMMA), and model groups, respectively.

All RNA samples exhibited high integrity (RIN: 8.6 ± 0.5, range 7.8–9.5). Sequencing generated an average of 25 million paired-end 150-bp reads per sample, with post-trimming read lengths of 145 ± 3 bp and adapter contamination rates < 0.5%. Analysis revealed that all genes (100%) had quality scores > 35, with error rates < 0.1% (Fig 2A, B). Alignments between each sample and the reference genome was > 90% (except for C1 at 86.38%) (Table 2). These metrics confirmed the suitability of the data for downstream analysis. PCA was then used to model and visualize the dispersion of samples among the normal, model, PMMA, and ES-PMMA groups based on transcriptomic sequencing data. As shown in Fig 2C, no strong outliers were identified. PC1 and PC2 explained 45.22% and 8.85% of the total variance, respectively, with a cumulative contribution of 54.07%. These results indicated that the sequencing data of all samples were of good quality.

**Table 2:** Control group (n = 5): Only the skin and subcutaneous tissue of the distal femur were incised and sutured.

Group	Sample	Mapping Rate	
Enoxaparin Sodium-Polymethylmethacrylate (ES-PMMA)	A1	90.22%	92.93% ± 1.46%
	A2	90.91%	
	A3	92.45%	
	A4	94.18%	
	A5	93.33%	
	A6	93.84%	
	A7	92.83%	
	A8	94.83%	
	A9	92.05%	
	A10	94.61%	
Control	B1	95.47%	94.32% ± 0.80%
	B2	94.46%	
	B3	94.16%	
	B4	92.97%	
	B5	94.53%	
Polymethylmethacrylate (PMMA)	C1	86.38%	93.11% ± 2.65%
	C2	94.85%	
	C3	90.66%	
	C4	95.03%	
	C5	95.21%	
	C6	93.00%	
	C7	92.27%	
	C8	94.84%	
	C9	94.78%	
	C10	94.12%	
Model	D1	94.66%	94.31% ± 0.96%
	D2	94.24%	
	D3	95.80%	
	D4	94.80%	
	D5	94.42%	
	D6	95.42%	
	D7	93.50%	
	D8	94.53%	
	D9	92.98%	
	D10	92.76%	

Model group (n = 10): A 3 mm × 1 cm bone defect was created in the distal femur; PMMA (Polymethylmethacrylate) group (n = 10): Polymethyl methacrylate was implanted into the bone defect; ES-PMMA (Enoxaparin Sodium-Polymethylmethacrylate) group (n = 10): Modified ES-PMMA material was implanted into the bone defect. Alignment between the sample and the reference genome (> 90%). Group represents the grouping information. Sample represents the sample name and Mapping Rate represents the comparison rate. Hisat2 software (version 2.2.1) (using default parameters) was used for alignment analysis against the human reference genome (GRCh37). Filtering criteria were as follows: using a sliding window (4 bp window, average quality ≥ 20) to cut off low-quality regions, removing bases at the beginning and end with quality values < 3, removing adapter sequences by ILLUMINACLIP (2 mismatches/ score threshold 30), and discarding reads < 36 bp.

### 3.2. GSVA revealed the top 10 pathways that were activated or inhibited

**Fig 3:**
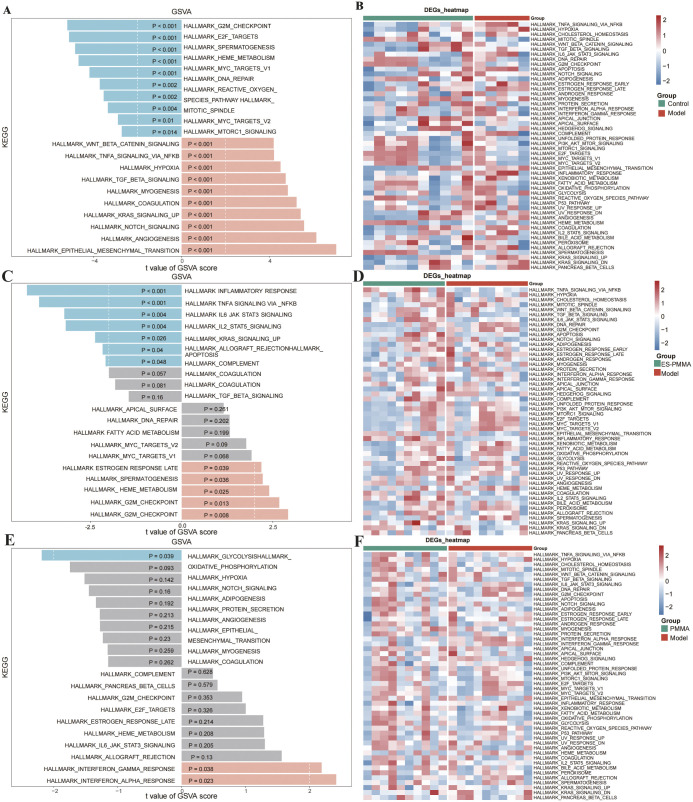
GSVA (Gene Set Variation Analysis) analysis of pathway activity. (A-B) Model vs. Control: Top 10 pathways show upregulated inflammation and suppressed proliferation. (C-D) PMMA vs. Model: Upregulated inflammation and mixed proliferation effects. (E-F) ES-PMMA vs. Model: Suppressed inflammation and enhanced proliferation. Barplots show enrichment t-scores; heatmaps display pathway enrichment. Blue representing inhibition and orange representing activation.

GSVA was performed to evaluate pathway activity between groups. The reference gene set was the “h.all.v7.4.symbols.gmt” gene set obtained from the MSigDB database. Permutation number was 100 times and FDR-adjusted p-values < 0.05 were considered significant pathways. The FPKM data of gene expression was first converted from the original counts to FPKM values through the count ToFpkm function. Subsequently, a logarithmic transformation (log2 (FPKM + 1)) was applied for normalization prior to GSVA analysis. Pathways with |NES| > 1 were presented. When comparing the model group and the control group, 20 pathways were activated, and 11 pathways were inhibited. When comparing the PMMA and model groups, 17 pathways were activated, and 24 pathways were inhibited. When comparing the PMMA and control groups, 23 pathways were activated, and 28 pathways were inhibited. Therefore, we selected the top 10 pathways for further analysis. When comparing the ES-PMMA and model groups, analysis revealed that a range of inflammation-related pathways, among which TNF-α signaling *via* NF-KB (adj.p = 0.017, NES = −0.39) and inflammatory response (adj.p = 0.014, NES = −0.42), were the most significant pathways, thus indicating that ES-PMMA successfully alleviated inflammation. In contrast, the E2F target pathway (adj.p = 0.07, NES = 0.55) regulates cell cycle progression, the G2M checkpoint pathway (adj.p = 0.11, NES = 0.47) controls mitosis, and the HEME metabolism pathway (adj.p = 0.16, NES = 0.37) governs heme biosynthesis. Metabolism-related pathways included spermatogenesis (adj.p = 0.18, NES = 0.19) and estrogen response late (adj.p = 0.18, NES = 0.14). All of these pathways are critical for cell proliferation and differentiation. Although the adjusted p-value exceeded 0.05, these findings still suggest/indicate the potential of ES-PMMA to promote cell proliferation and differentiation (Fig 3A, B). When comparing the PMMA and model groups, the inflammation-related pathways, including the IFN-α response (adj.p = 0.64) and IFN-γ response (adj.p = 0.64), showed no statistically significant differences. This suggests that, at the selected time point (10 days post-implantation), PMMA did not induce a significant inflammatory perturbation in the bone defect tissue compared to the model group (Fig 3C, D). When comparing the model and control group, epithelial-mesenchymal transition (EMT) (adj.p = 7.52e-05, NES = 0.39), angiogenesis (adj.p = 0.0002, NES = 0.20), Notch signaling (adj.p = 0.0004,NES = 0.11), Kras signaling-up (adj.p = 0.0005, NES = 0.21), coagulation myogenesis (adj.p = 0.0005, NES = 0.19), TGF-β signaling (adj.p = 0.0008, NES = 0.8), TNF-α signaling via NF-κβ (adj.p = 0.0018, NES = 0.32) and the WNT-β-catenin (adj.p = 0.0018, NES = −0.04) signaling pathways were activated. However, the Mtorc1 signaling (adj.p = 0.0306, NES = −0.08), Myc target v2 (adj.p = 0.0221, NES = −0.24), mitotic spindle (adj.p = 0.0090, NES = −0.34), reactive oxygen species (ROS) (adj.p = 0.0062, NES = −0.10), DNA repair (adj.p = 0.0044, NES = −0.20), Myc target v1 (adj.p = 0.0018, NES = −0.26), HEME metabolism (adj.p = 0.0008, NES = −0.12), spermatogenesis (adj.p = 0.0007, NES = −0.12), E2F target (adj.p = 0.0005, NES = −0.25), and G2M checkpoint (adj.p = 0.0005, NES = −0.27) pathways were all inhibited, thus indicating that cell proliferation and differentiation had been inhibited (Fig 3E, F). Collectively, these results indicated that cell proliferation and differentiation were restricted during inflammation, at least to some extent.

### 3.3. Inter-group differences and the identification of key candidate genes associated with ES-PMMA

#### 3.3.1. Screening for DEGS.

**Fig 4:**
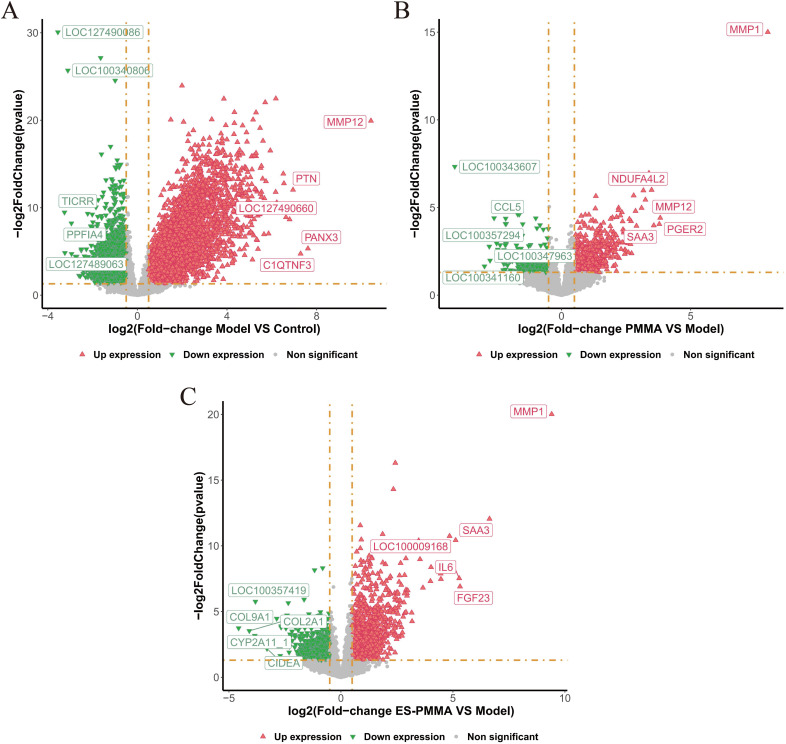
Differential gene expression between groups. (A) A total of 6637 DEGs, including 3871 up-regulated and 2766 down-regulated genes, were identified between the model and control groups. (B) A total of 1071 DEGs, including 713 up-regulated genes and 358 down-regulated genes, were identified between the PMMA and model groups. (C) A total of 2059 DEGs, including 1239 up-regulated and 820 down-regulated genes, were identified between the ES-PMMA and model groups. Green representing inhibition and red representing activation.

A total of 6637 DEGs, including 3871 up-regulated and 2766 down-regulated genes, were identified between the model and control groups. A total of 1071 DEGs, including 713 up-regulated genes and 358 down-regulated genes, were identified between the PMMA and model groups. A total of 2059 DEGs, including 1239 up-regulated and 820 down-regulated genes, were identified between the model and ES-PMMA groups. The DEGs identified between the model and control groups, the PMMA and model groups, and the ES-PMMA and model groups, were named DEG-1, DEG-2, and DEG-3, respectively. Volcano plots demonstrating these DEGs are shown in Fig 4A–C with a screening threshold of adjusted p-value (FDR) < 0.05 and |log2FC| > 0.5.

#### 3.3.2. Screening of key candidate genes in ES-PMMA bone cement.

**Fig 5:**
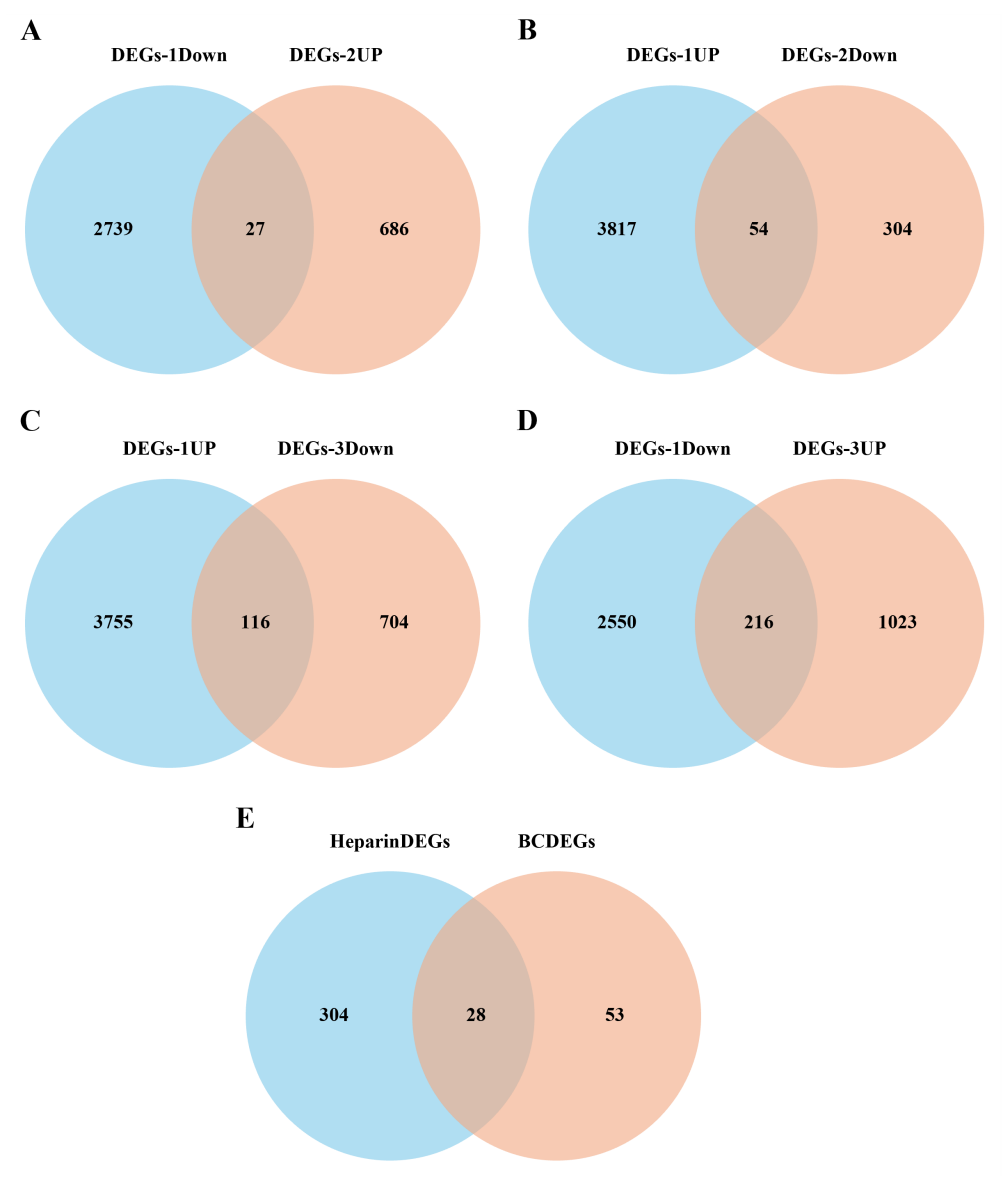
Screening of key candidate genes associated with ES-PMMA. (A, B) Identification of bone cement-associated differentially expressed genes (DEGs). Venn diagram was used to obtain the genes with opposite expression trends (81 genes) in DEGs-1 and DEGs-2, which was defined as bone cement-associated DEGs. (C, D) Identification of heparin-cement co-regulated DEGs. Venn diagram was used again to obtain the genes with opposite expression trends in DEGs-1 and DEGs-3(332 genes in total), which was defined as heparin-cement co-regulated DEGs. (E) Selection of heparin-cement signature genes. A 28-gene overlap subset was identified between heparin-cement co-regulated DEGs and cement-associated DEGs. Subtraction of this overlapping subset from the 332 heparin-cement co-regulated DEGs yielded 304 heparin-cement signature genes.

A Venn diagram was used to identify genes with opposing expression trends in DEGs-1 and DEGs-2. We took the intersection of the up-regulated genes in DEGs-1 and the down-regulated genes in DEGs-2, and the intersection of the down-regulated genes in DEGs-1 and the up-regulated genes in DEGs-2, and then identified the union of the two intersections. We obtained 81 genes which were defined as bone cement-related DEGs (Fig 5A, B). Similarly, a Venn diagram was used to identify genes with opposing expression trends in DEGs-1 and DEGs-3 by taking the intersection of the up-regulated genes in DEGs-1 and the down-regulated genes in DEGs-3, and the intersection of the down-regulated genes in DEGs-1 and the up-regulated genes in DEGs-3, and then identifying the union of the two intersections. We identified 332 genes which were defined as heparin bone cement-related DEGs (Fig 5C, D). A 28-gene overlap subset was identified between heparin-cement co-regulated DEGs and cement-associated DEGs. Subtraction of this overlapping subset from the 332 heparin-cement co-regulated DEGs yielded 304 heparin-cement signature genes (Fig 5E).

### 3.4. Enrichment analysis

#### 3.4.1. Gene Ontology (GO) pathway enrichment analysis.

**Fig 6:**
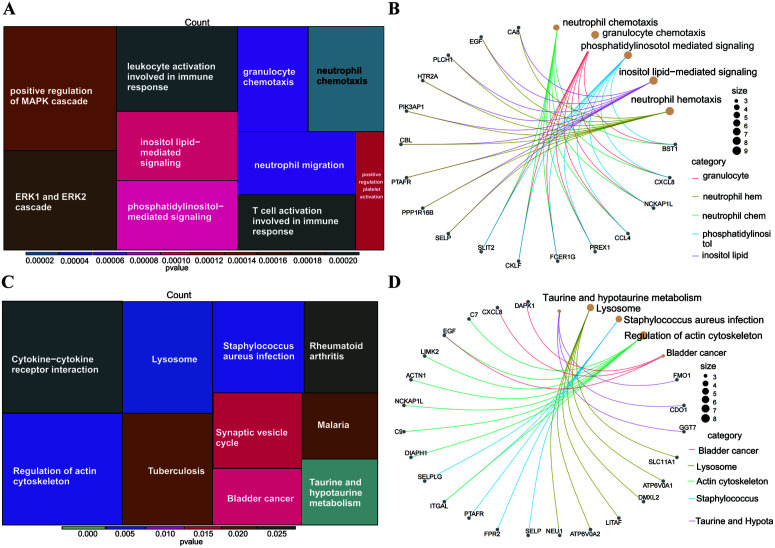
Gene Ontology (GO) and Kyoto Encyclopedia of Genes and Genomes (KEGG) enrichment analyses of key candidate genes associated with ES-PMMA. (A, B). Results of GO enrichment analysis; (C, D). Results of KEGG enrichment analysis. Each module represents each pathway, the size of the module represents the level of gene expression in the pathway, and the color represents the P-value.

GO analysis revealed that the main enriched terms (FDR < 0.05) included response to lipopolysaccharides, response to molecules of bacterial origin, and cell-substrate adhesion (cell-matrix adhesion). The key candidate genes associated with ES-PMMA were enriched in 468 terms; of these, 350 terms were related to biological processes, 55 terms were related to cellular components, and 63 terms were related to molecular functions. The main enriched terms included tertiary granule (12/193), secretory granule membrane (14/193) and specific granule (10/193) (Fig 6A, B).

#### 3.4.2. Kyoto Encyclopedia of Genes and Genomes (KEGG) pathway enrichment analysis.

KEGG analysis key candidate genes associated with ES-PMMA were significantly enriched in 18 pathways, including taurine and hypotaurine metabolism (3/104), lysosome (6/104), and Staphylococcus aureus infection pathways (5/104) (Fig 6C, D). Taurine metabolism synergistically enhances the immune regulatory function of lysosomes through antioxidant, anti-inflammatory and osteogenic pathways, thereby jointly optimizing the bone integration and anti-inflammatory microenvironment, which may represent the key advantage of ES-PMMA bone cement.

### 3.5. Candidate genes identified by PPI network analysis

**Fig 7:**
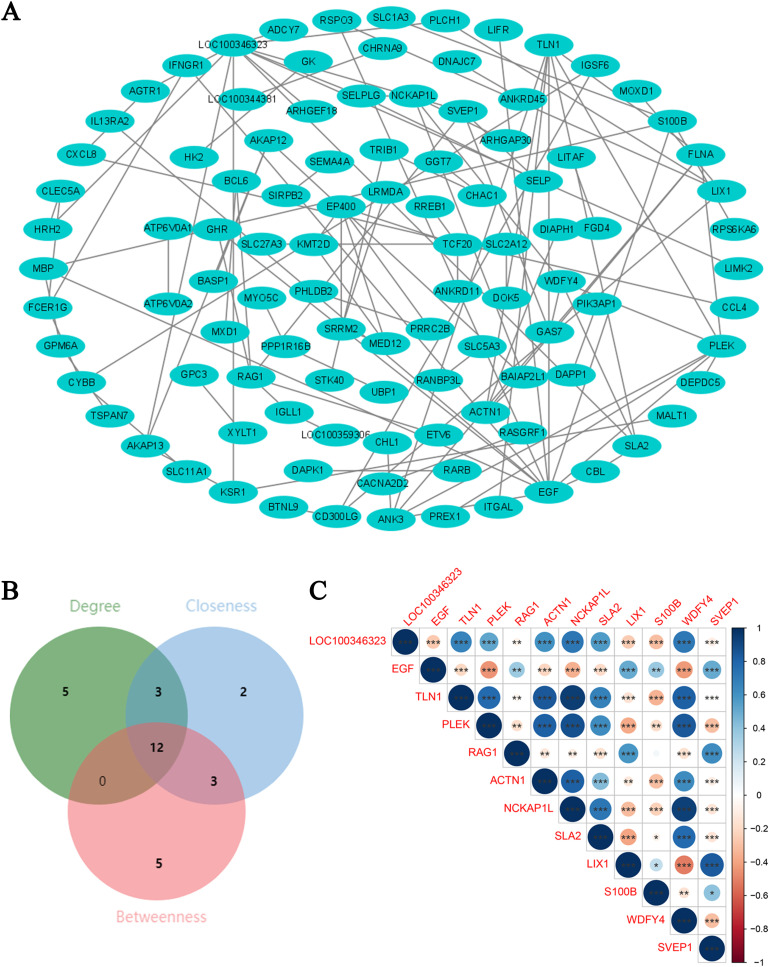
Screening of candidate genes via protein–protein interaction (PPI) network analysis and subsequent correlation analysis. A. A PPI network was constructed to investigate the interaction between the key candidate genes associated with Enoxaparin Sodium Polymethyl methacrylate (ES-PMMA); B. The top 20 genes identified using the Degree, Closeness, and Betweenness algorithms each were intersected, and 12 candidate genes were selected; C. Correlation analysis of the 12 candidate genes. Blue represents a positive correlation, and red represents a negative correlation. The larger the correlation, the larger the circle and the darker the color. ‘*’ represents statistical significance.

The PPI network was visualized by Cytoscape software and featured 103 nodes and 108 edges, representing the intricate web of interactions between these proteins within a biological system (Fig 7A). Data acquired from the STRING database were imported into Cytoscape, and genes were scored by the Degree, Closeness and Betweenness algorithms in the CytoHubba plug-in. The top 20 genes identified by each algorithm were intersected, resulting in the identification of 12 candidate genes (Fig 7B). Spearman’s correlation analysis was then performed to investigate the relationship between candidate genes in the transcriptomic sequencing dataset of the ES-PMMA and the model groups (|cor| > 0.3 and P < 0.05). Analysis revealed that Talin 1 (*TLN1*) and *NCK*-associated protein 1-like (*NCKAP1L*) exhibited the most significant positive correlation, whereas *LIX1* and *WDFY* family member 4 (*WDFY4*) exhibited the most significant negative correlation (Fig 7C). TLN1, which mediates cytoskeletal linkage, and NCKAP1L, which regulates immune cell motility, may synergize to enhance immune cell adhesion and migration, potentially supporting the immunomodulatory effects of ES-PMMA during bone repair.

### 3.6. Machine learning

**Fig 8:**
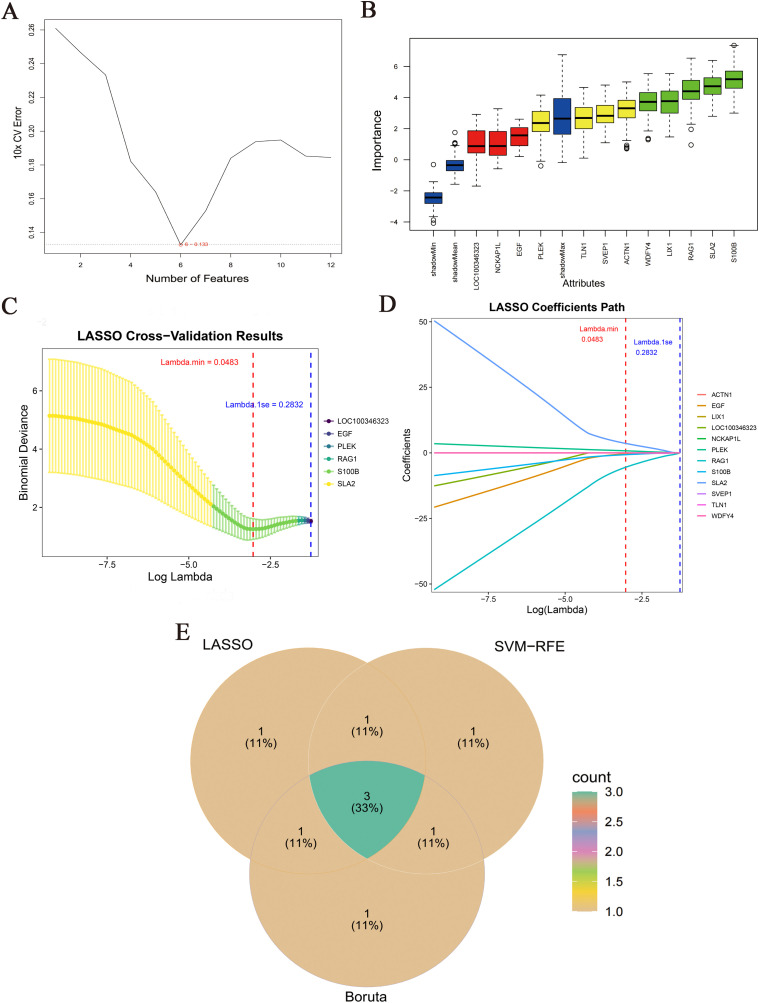
Candidate genes were analyzed by three machine learning algorithms: LASSO regression, support vector machine recursive feature elimination (SVM-RFE), and Boruta. (A) SVM-RFE analysis identified six signature genes; (B) Boruta analysis identified five signature genes; (C, D) LASSO analysis identified five signature genes (lambda. min = 0.0162); E. Three key genes (RAG1, SLA2, and S100B) were identified after the three gene sets were intersected.

Next, we selected key genes from the 12 candidate genes associated with the ES-PMMA and model groups identified in Section 3.5. We employed a triple machine learning algorithm to screen key genes, as follows. SVM-RFE was performed using the R package “e1071” and parameter performance was evaluated by 10-fold cross-validation. The recursive elimination process was initiated with all candidate genes and involved three core steps: (1) training SVM models with optimal parameters, (2) computing feature weights, (3) eliminating the lowest ranked 10% of features. Termination criteria required a > 0.005 increase in average cross-validation error for three consecutive iterations. To enhance stability, external 3-fold cross-validation was repeated 10 times. Genes with a selection frequency > 0.8 (selected occurrences/total runs) were retained, yielding six feature genes (Fig 8A).

Boruta analysis was employed to perform random forest-based permutation tests and retain genes with importance scores significantly higher than synthetic noise features. We calculated the Z-score (importance score) for each real gene and shadow feature. Iterations continued until all features attained definitive classifications. When the feature Z value was greater than the maximum shadow feature Z value and p < 0.01, the condition was marked as “Confirmed” (a significant important feature) resulting in five confirmed feature genes (Fig 8B).

Next, LASSO logistic regression was implemented via the “glmnet” package. The regularization path comprised 100 exponentially decreasing λ values with an elastic net parameter α = 1 (pure LASSO). The optimal λ was selected by 10-fold cross-validation (λ = 0.0162). Features with non-zero coefficients (|β| > 0) were retained, identifying five genes (Fig 8C, D). Integration of the three machine learning outcomes followed strict criteria: only genes concurrently selected by SVM-RFE, Boruta, and LASSO were retained. Three key genes (*RAG1*, *SLA2*, *S100B*) were ultimately identified (Fig 8E).

### 3.7. ROC analysis and evaluation

**Fig 9:**
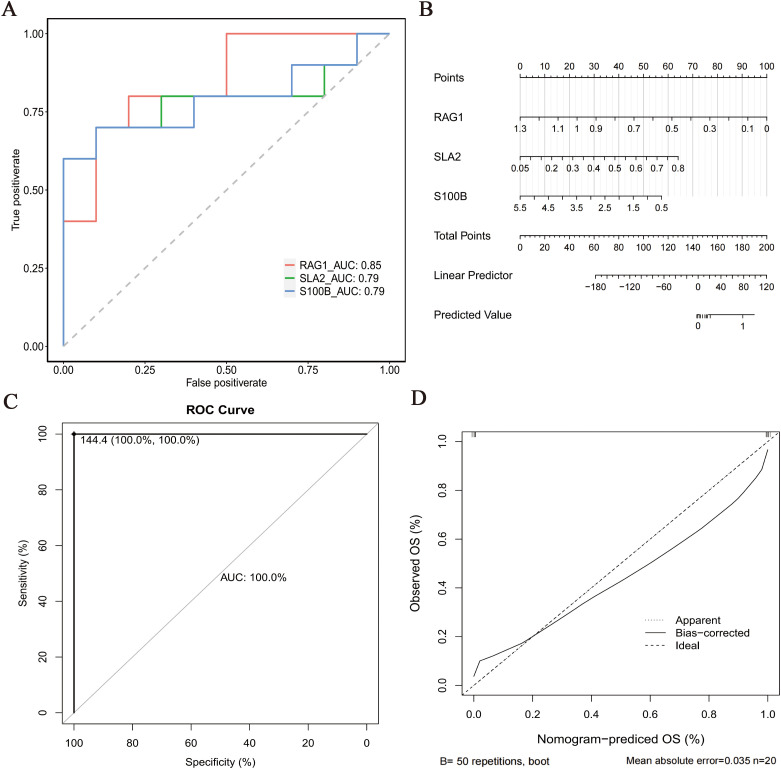
Assessment of key genes. A. The receiver operating characteristic analysis (pROC). package was used to generate ROC curves for the three key genes. Area Under the Curve (AUC) values of > 0.7 indicated that the genes had good discrimination performance; B. The rms package was used to construct a nomogram model for risk prediction in the Enoxaparin Sodium-Loaded Polymethylmethacrylate (ES-PMMA) versus the model group based on the three biomarkers; C. ROC analysis (AUC > 0.7) was performed to evaluate the predictive performance of the nomogram, with a result of 1, indicating good performance; In this curve, the abscissa represented the predicted probability, whereas the ordinate represented the actual probability. D. Calibration curve of the nomogram. The slope of the curve was close to 1, validating the predictive accuracy of the nomogram.

Our comprehensive analysis demonstrated outstanding predictive performance across all evaluation metrics. The machine learning models achieved exceptional classification: LASSO (accuracy = 1.00, recall = 1.00, AUC-ROC = 1.00), SVM (accuracy = 0.909, recall = 1.00, AUC-ROC = 1.00), and Boruta (accuracy = 1.00, recall = 1.00, AUC-ROC = 1.00) all showed perfect or near-perfect discrimination (Figure S1 A-F in S1 File). Next, the pROC package in RStudio was used to generate ROC curves for the three key genes using the 70:30 training: test split established in the machine learning phase. The AUC confidence intervals were as follows: *RAG1* (0.6784–1), *SLA2* (0.562–1) and *S100B* (0.5677–1), all significantly >0.7 (DeLong’s test, all p < 0.001), indicating their robust discriminatory ability between the ES-PMMA and model groups (Fig 9A). These genes were consequently selected as potential biomarkers. ROC analysis of the nomogram demonstrated perfect discrimination (AUC = 1.00, 95% CI: 1.00–1.00) in predicting risk stratification between groups (Fig 9B). In terms of calibration metrics, the Hosmer-Leme test (10 bins) (χ² = 40,043,602.04, df = 4, p < 2e-16, p ≤ 0.05) indicated significant calibration bias) while calibration curve analysis (slope = 0.98, 95% CI: 0.95–1.01) confirmed excellent agreement between predicted and observed outcomes (Fig 9C, D), thus validating the predictive accuracy of the nomogram.

### 3.8. Differential expression of candidate biomarkers

**Fig 10:**
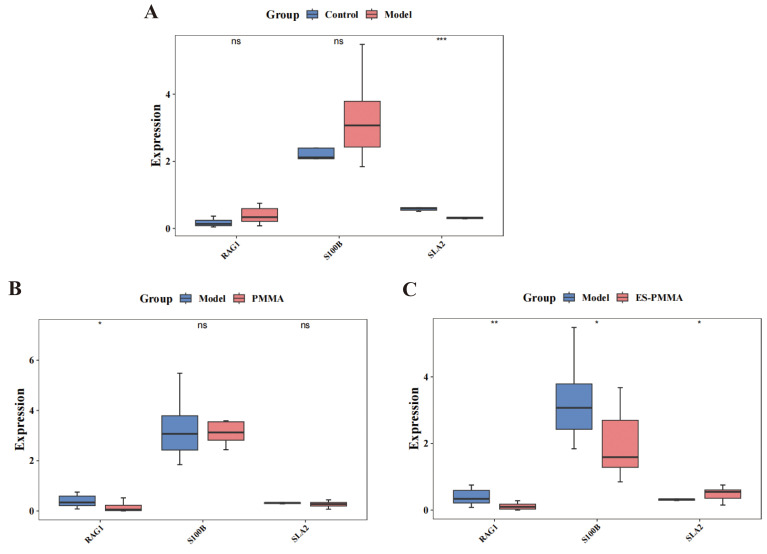
Gene expression analysis of Src-like adaptor 2 (*SLA2*), Recombination activating gene 1 (*RAG1*) and S100 calcium-binding protein B(*S100B*). A. Control Group vs. Model Group: *RAG1* and *S100B* showed high expression, while *SLA2* showed low expression. B. PMMA Group vs. Model Group: *RAG1* exhibited low expression, whereas *S100B* and *SLA2* showed no significant differences in expression levels. C. ES-PMMA Group vs. Model Group: *RAG1* and *S100B* demonstrated low expression, while *SLA2* showed high expression. (Mann-Whitney U test, * *P* < 0.5; **** *P* < 0.001).

Quantitative analysis revealed statistically significant alterations in *RAG1*, *S100B*, and *SLA2* expression across experimental groups. One-way ANOVA was used to conduct an overall difference test on multiple groups, and then pairwise comparisons between groups were performed using Tukey’s post-hoc test. (FDR-adjusted p < 0.05 for all comparisons). Compared to the control group, the expression of *RAG1* and *S100B* in the model group was higher but with no significant difference, while *SLA2* was significantly reduced (p = 0.007), thus suggesting that the inflammatory response period following bone injury persisted and had gradually stabilized (Fig 10A). Compared to the model group, the expression of *RAG1* in PMMA group decreased mildly (p = 0.0232). Furthermore, the expression level of *RAG1* was very low in both groups (FPKM<1), and there was no statistically significant difference in the expression levels of *S100B* and *SLA2*. Therefore, it can be concluded that PMMA had no significant effect on the inflammatory response after bone injury (Fig 10B). When compared to the model group, the expression levels of *RAG1* and *S100B* in the ES-PMMA group were significantly lower (p = 0.0067 and p = 0.0288), while the expression levels of *SLA2* were significantly higher (p = 0.0288). These directional reversals suggest that ES-mediated anti-inflammatory effects occurred through the suppression of *RAG1*/ *S100B* and the activation of *SLA2* (Fig 10C).

### 3.9. TF prediction by the three key genes

**Fig 11:**
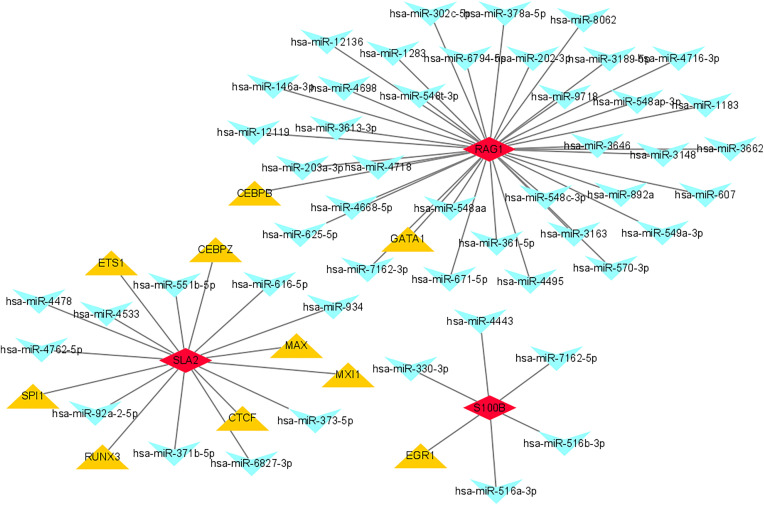
Computational interrogation identified upstream TFs (predicted via ChEA3 using ENCODE ChIP-seq, ReMAP, and de novo motif screening; NES > 3.0) and miRNAs (miRDB; Target score ≥ 85, 80% validation accuracy). The integrated network (Cytoscape 3.9.1) comprised 63 nodes (TFs, biomarkers, miRNAs) and 60 confidence-weighted edges, with SP1 (RAG1-associated TF) and miR-26b-5p (top RAG1 miRNA, score = 97) highlighted as central hubs.

Computational interrogation of upstream regulatory mechanisms identified both transcription factors (TFs) and miRNAs governing key biomarkers. Using stringent ChEA3 analysis (integrating ENCODE ChIP-seq, ReMAP, and *de novo* motif screening; NES > 3.0 threshold), we predicted high-confidence TFs for: *RAG1* (2 TFs), *S100B* (1 TF), and *SLA2* (7 TFs). Parallel miRDB analysis (Target score ≥ 85, 80% validation accuracy) revealed miRNA regulators: 35 targeting *RAG1*, 5 for *S100B*, and 10 for *SLA2*. Integration of these predictions generated a TF-biomarker-miRNA network (Cytoscape 3.9.1) comprising 63 nodes and 60 confidence-weighted edges, highlighting SP1 (*RAG1*-associated TF) and miR-26b-5p (top-scoring *RAG1* miRNA; score = 97) as central regulatory hubs (Fig 11).

### 3.10. RT-qPCR Validation for Key Genes (*SLA2*, *RAG1*, *S100B*)

For *S100B* expression, the ES-PMMA Groups showed significantly reduced expression versus both PMMA (mean difference 0.27, [0.1412, 0.3988], p < 0.0001) and Model groups (mean difference 0.2000, [0.07123, 0.3288], p = 0.0016), thus confirming their anti-inflammatory potency (Figure S2A in S1 File). For *SLA2* expression, both the model group and PMMA group showed marked suppression when compared to the control group (mean difference 0.3700, [0.1474,0.5926], p = 0.0008), (mean difference 0.4300, [0.2074,0.6526], p = 0.0001), while ES-PMMA partially reversed this effect (PMMA vs ES-PMMA: −0.2900, [−0.5126, −0.06740], p = 0.008) (Figure S2B in S1 File). *RAG1* expression patterns further supported the immunomodulatory properties of ES-PMMA, with significant reductions when compared to the PMMA and Model groups (mean difference 0.1500, [0.008430,0.2916], p = 0.0354), (mean difference 0.1800, [0.3843,0.3216], p = 0.0098) (Figure S2C in S1 File). Consistent statistical significance across multiple comparison methods confirmed the anti-inflammatory benefits of ES conjugation to PMMA.

## 4. Discussion

Previous studies focused on the bone immune microenvironment have yielded novel insights into bone repair mechanisms and bone homeostasis during inflammation. The bone immune microenvironment plays an important role in regulating the repair process following bone trauma. Bone implant materials should ideally exert immunoregulatory effects to promote tissue repair. Consequently, research studies are gradually shifting their focus from the ‘biological inertia’ to the ‘biological activity’ of implant materials [39,40]. In this study, we performed transcriptomic sequencing on tissue samples from a rabbit model of bone defects to identify the genes and mechanisms by which ES, as an additive for bone implant materials, regulates bone repair in the immune microenvironment.

PMMA, is widely used in clinical practice owing to its excellent performance; however, this material cannot meet the specific requirements of contemporary clinical medicine owing to its cytotoxicity, ability to induce inflammatory responses and poor biocompatibility. Furthermore, as a foreign body, PMMA can trigger an inflammatory response. In previous research, Ji et al. identified that the inflammatory factor TNF-α induced by PMMA mediated the apoptosis of surrounding cells, whereas its antagonist reversed this pro-apoptotic effect [41]. In another study, Mideiros et al. found that osteonecrosis and inflammatory reaction at the PMMA-bone interface could last for over eight weeks, as demonstrated by histological analysis [42]. Apoptosis and inflammation are known to be closely related. Tatsumi et al. found that the apoptosis of osteocytes accelerated the differentiation of osteoclasts and aggravated bone resorption [43]. Although moderate inflammatory responses play an important role in bone repair, the excessive or long-term activation of inflammation can impede bone repair [44,45]. Therefore, we incorporated ES into PMMA and aimed to investigate the impact of ES on bone tissue inflammatory responses by examining its ability to enhance the biocompatibility of PMMA. When compared to PMMA, our previous research showed that ES-PMMA significantly suppressed the apoptosis of RAW264.7 cells and reduced the release of inflammatory factors induced by lipopolysaccharide (LPS) in animal models of joint replacement and bone defects [32,36]. Furthermore, research has demonstrated that ES possesses the ability to upregulate the expression of CD206 in M1 macrophages [36].

In the present study, transcriptomic sequencing of tissue samples from rabbits with bone defects, combined with Gene Set Variation Analysis (GSVA), revealed that in inflammation-related pathways, the expression levels of TNF-α and TGF-β were significantly higher in the model group than in the control group. TNFα is secreted primarily by monocytes and macrophages and promotes the secretion of IL-1 and other inflammatory factors, thereby amplifying the inflammatory response [46]. TGFβ is secreted by monocytes, macrophages and lymphocytes and plays an important role in regulating inflammation and bone repair [47]. The high expression levels of TNFα and TGFβ in the model group indicated that rabbits with bone defects were suffering from inflammation when the samples were acquired. When comparing the PMMA and model groups, except for the increased expression levels of IFN-γ and IFN-α signaling pathways, there was no significant difference in other signaling pathways. Considering that IFN-γ and IFN-α are pro-inflammatory factors, PMMA can exert certain pro-inflammatory and inflammatory effects on osteogenesis [48]. Therefore, these data suggest that the biological activity of PMMA is poor, and its immunomodulatory effects on bone repair are limited. Compared with the model group, the ES-PMMA group exhibited a lower extent of IL-2 signaling, IL-6 signaling, and both inflammatory and apoptotic pathways. IL-2 signaling, IL-6 signaling, and inflammatory pathways promote inflammatory responses, and the apoptosis pathway is associated with inflammation [49,50]. Furthermore, the G2M checkpoint, E2F target, and other signaling pathways, which are all related to cell proliferation and differentiation, were activated in the ES-PMMA group [51,52]. Consistent with our previous studies [32,36], the findings of the present study suggest that ES-PMMA exerts both anti-inflammatory and anti-apoptotic effects during bone repair.

A total of 332 dominant DEGs associated with ES-PMMA were identified in this study. GO analysis revealed that these genes were predominantly enriched in the tertiary granule pathway and other pathways involving the components and functions of leukocytes. KEGG analysis further revealed that these genes were predominantly enriched in pathways related to inflammation and cellular metabolism, including taurine and hypotaurine metabolism and *Staphylococcus aureus* infection. Taurine and hypotaurine metabolism have both been associated with inflammatory responses [53]. The integrated machine learning framework leveraged complementary algorithmic strengths for robust feature selection: SVM-RFE optimized feature discriminability through hyperplane margin maximization. The advantage of SVM-RFE is that it is adept at handling small sample high-dimensional data and can eliminate irrelevant features recursively. Boruta provided statistical robustness against random fluctuations via shadow attribute comparisons, while LASSO enforced feature space sparsification through L1 regularization. The advantage of choosing LASSO is that it automatically sparsifies features by L1 regularization, and outputs interpretable feature coefficients; this is suitable for feature selection in high-dimensional data (such as gene expression data). This synergistic approach minimized false positives while identifying high-confidence biomarkers. The computational pipeline ultimately revealed three key mediators of ES-PMMA interactions: *SLA2* (Src-like adaptor 2), *RAG1* (Recombination activating gene 1), and *S100B* (S100 calcium-binding protein B), demonstrating the power of combined feature selection methodologies in the discovery of translational biomarkers. *S100B* and *RAG1* were both downregulated in the ES-PMMA group but significantly upregulated in the PMMA and model groups. In contrast, the expression levels of *SLA2* were significantly higher in the ES-PMMA group than in the PMMA and model groups. *RAG1* encodes the RAG1 protein, which forms a tetrameric RAG complex with RAG2 protein. The recombination of variable (V), diversity (D), and joining (J) genes leads to the production of Ig and T cell receptor (TCR), which form the basis of antigen recognition during early T- and B-cell development and maturation [54]. *RAG1* is known to be a key regulatory gene involved in the maturation of T and B cells in developing lymphocytes and an important component of the adaptive immune system. In *RAG1*/*RAG2*-deficient animal models, T- and B-cell maturation has been shown to be inhibited [55]. In addition, patients with abnormal RAG1 expression may develop severe immunodeficiency disorders, such as Omenn syndrome [56,57].

SLA2 encodes the SLAP2 protein, which is a coding adaptor protein belonging to the SLAP family. This gene is expressed in various cells; however, previous research on SLA2 predominantly focused on T-cell immune responses [58,59]. SLA2 may function by linking the zeta chain of T-cell receptor-associated protein kinase 70 (ZAP70) and other signaling proteins to Casitas B lineage lymphoma proto-oncogene (CBL), leading to CBL-dependent protein degradation [60]. The overexpression of *SLA2* is known to suppress the transcription of genes mediated by nuclear factor of activated T cells (NFAT), activator protein 1 and IL-2. Therefore, SLAP acts as a negative regulator of TCR complex-mediated signaling and can attenuate T cell-mediated adaptive immune responses [61]. The role of SLA2 has been extensively investigated in the context of viral and neoplastic diseases [62]. The effects of SLA2 on T-cell immune responses are opposite to those of RAG1; this is consistent with their different expression patterns in the ES-PMMA group in this study. RAG1 and SLA2 may play an immunomodulatory role in the skeletal system by regulating T/B-cell-mediated adaptive immune responses.

S100B, a member of the S100 family encoded by the *S100B* gene, is a calcium-binding protein containing two EF-hand motifs, which are helix-loop-helix structures that bind calcium ions to regulate protein function. S100 proteins are localized in the cytoplasm and/or nucleus of various cells and participate in the regulation of cellular calcium homeostasis, enzyme activity, cytoskeleton interactions, cell survival, cell differentiation and cell proliferation [63,64]. S100B is involved in various immune-related pathways, including the IL-1, Toll-like receptor and NF-κβ signaling pathways. Furthermore, S100B is expressed in T cells, B cells and macrophages. Extracellular S100B is thought to interact mainly (but not exclusively) with target cells *via* receptor for advanced glycation end products (RAGE), a multi-ligand transmembrane receptor, regulating signaling cascades under physiological conditions at low molar concentrations and directly acting as a damage-associated molecular pattern (DAMP) protein (a pathogenic factor) at high micromolar concentrations [65]. S100B is predominantly secreted by astrocytes in the nervous system and may be involved in the pathological mechanisms of neurodegenerative diseases, such as Alzheimer’s disease (AD) and Parkinson’s disease (PD) [66,67]. S100B levels are known to be elevated in various biological fluids, including cerebrospinal fluid, peripheral and umbilical cord blood, amniotic fluid, saliva, urine and feces. The S100B protein is considered an important biomarker for monitoring disease status and represents a promising therapeutic target. The inhibition of S100B has been shown to prevent the development of inflammation and alleviate disease symptoms [68]. Although the functions and cellular localization of S100B in the nervous system have been extensively investigated, the precise functional role of S100B in non-neural locations has received far less attention. To the best of our knowledge, this is the first study to report the abnormal expression of *S100B* in an animal model of bone injury. We previously demonstrated that ES promoted the LPS-induced polarization of RAW264.7 cells to M2 macrophages *in vitro* [36], whereas S100B inhibited the anti-inflammatory and neuroprotective M2 polarization and promoted M1 polarization in Middle Cerebral Artery Occlusion (MCAO) mouse models *in vivo* [68]. In the present study we revealed that ES inhibited the expression of *S100B*, thus enhancing our understanding of the mechanism by which ES suppresses inflammatory responses.

While this study provided insights into the mechanism of ES in regulating the immune microenvironment during bone repair, there are certain limitations to our study that need to be considered. First, the tissue samples used in this study were collected from rabbits with femoral bone defects. The mechanism by which ES regulates bone wound repair in rabbits may differ from that in humans. Second, considering that bone repair is a dynamic regulatory process, the inflammatory response and bone repair effects are likely to differ at different stages. Therefore, samples collected at different time points may exhibit different gene expression patterns. The study collected samples at a single time point (10 days post-surgery), limiting insights into the temporal dynamics of acute, chronic, and repair phases of inflammation. Additionally, bioinformatics analyses, while robust, rely on statistical thresholds and may miss low-expression genes or context-specific interactions, requiring experimental validation. Third, this study did not address potential biological constraints of ES, such as dose-dependent effects or toxicity at high concentrations. Future studies should evaluate optimal ES dosages and long-term safety in bone repair applications.

## 5. Conclusion

In this study, we investigated the potential of ES as an additive to PMMA for bone repair. During the bone repair process, ES has been shown to effectively alleviate inflammatory responses *via* various mechanisms. Based on the findings of this study, ES holds significant promise as an additive for bone implant materials, with the potential to significantly enhance their biocompatibility and reduce inflammatory responses. To facilitate clinical translation, further experimental validation is now required for the application of ES in other bone repair materials, such as hydroxyapatite-based composites. Furthermore, there is as significant need to develop modified forms of heparin, based on the molecular structure of ES and designed to exhibit superior anti-inflammatory properties; these may represent highly suitable additives for bone materials.

## Supporting information

S1 File **Supplementary Figure 1. Precision-Recall and ROC Curves for LASSO, SVM, and Boruta Models.** (A, B) (LASSO Model): Accuracy = 1.0 (100%) | Recall = 1.0 (100%) | AUC-ROC = 1.0 (Perfect classification). (C, D) (SVM Model): Accuracy = 0.909 (90.9%) | Recall = 1.0 (100%) | AUC-ROC = 1.0 (Perfect classification). (E, F) (Boruta Model): Accuracy = 1.0 (100%) | Recall = 1.0 (100%) | AUC-ROC = 1.0 (Perfect classification). **Supplementary Figure 2. ES-PMMA exhibits anti-inflammatory effects by modulating *S100B*, *SLA2* and *RAG1* expression in validation experiments.** (A) *S100B* expression was significantly lower in ES-PMMA compared to PMMA (*p < 0.0001) and Model (p = 0.0016). (B) *SLA2* expression showed suppression in Model (*p = 0.0008) and PMMA (p = 0.0001) versus Control, with partial reversal by ES-PMMA (*p = 0.008 vs PMMA). (C) *RAG1* expression was reduced in ES-PMMA versus PMMA (*p = 0.035) and Model (p = 0.0098). Data shown as mean ± SEM (n = 6/group). One-way ANOVA with Tukey’s multiple comparisons test was used for all comparisons. *p < 0.05, **p < 0.01, ***p < 0.001.
(DOCX)

## References

[1] Horton JE, Oppenheim JJ, Mergenhagen SE, Raisz LG. Macrophage-lymphocyte synergy in the production of osteoclast activating factor. J Immunol. 1974;113(4):1278–87. 4414541

[2] Arron JR, Choi Y. Bone versus immune system. Nature. 2000;408(6812):535–6. doi: 10.1038/35046196 11117729

[3] Komatsu N, Takayanagi H. Mechanisms of joint destruction in rheumatoid arthritis - immune cell-fibroblast-bone interactions. Nat Rev Rheumatol. 2022;18(7):415–29. doi: 10.1038/s41584-022-00793-5 35705856

[4] Bozec A, Zaiss MM. T regulatory cells in bone remodelling. Curr Osteoporos Rep. 2017;15(3):121–5. doi: 10.1007/s11914-017-0356-1 28432597

[5] Pajarinen J, Lin T, Gibon E, Kohno Y, Maruyama M, Nathan K, et al. Mesenchymal stem cell-macrophage crosstalk and bone healing. Biomaterials. 2019;196:80–9. doi: 10.1016/j.biomaterials.2017.12.025 29329642 PMC6028312

[6] Shen B, Tasdogan A, Ubellacker JM, Zhang J, Nosyreva ED, Du L, et al. A mechanosensitive peri-arteriolar niche for osteogenesis and lymphopoiesis. Nature. 2021;591(7850):438–44. doi: 10.1038/s41586-021-03298-5 33627868 PMC7979521

[7] Haugen HJ, Lyngstadaas SP, Rossi F, Perale G. Bone grafts: which is the ideal biomaterial? J Clin Periodontol. 2019;46 Suppl 21:92–102. doi: 10.1111/jcpe.13058 30623986

[8] Choppadandi M, More N, Kapusetti G. Detoxification of poly(methyl methacrylate) bone cement by natural antioxidant intervention. J Biomed Mater Res A. 2019;107(12):2835–47. doi: 10.1002/jbm.a.36785 31433892

[9] Chiang CC, Hsieh MK, Wang CY, Tuan WH, Lai PL. Cytotoxicity and cell response of preosteoblast in calcium sulfate-augmented PMMA bone cement. Biomed Mater. 2021;16(5). doi: 10.1088/1748-605X/ac1ab5 34410226

[10] Khandaker M, Vaughan MB, Morris TL, White JJ, Meng Z. Effect of additive particles on mechanical, thermal, and cell functioning properties of poly(methyl methacrylate) cement. Int J Nanomed. 2014;9:2699–712. doi: 10.2147/IJN.S61964 24920906 PMC4043713

[11] Ricker A, Liu-Snyder P, Webster TJ. The influence of nano MgO and BaSO4 particle size additives on properties of PMMA bone cement. Int J Nanomed. 2008;3(1):125–32. 18488423 PMC2526357

[12] Pahlevanzadeh F, Bakhsheshi-Rad HR, Hamzah E. In-vitro biocompatibility, bioactivity, and mechanical strength of PMMA-PCL polymer containing fluorapatite and graphene oxide bone cements. J Mech Behav Biomed Mater. 2018;82:257–67. doi: 10.1016/j.jmbbm.2018.03.016 29627737

[13] Lv Y, Li A, Zhou F, Pan X, Liang F, Qu X, et al. A novel composite PMMA-based bone cement with reduced potential for thermal necrosis. ACS Appl Mater Interfaces. 2015;7(21):11280–5. doi: 10.1021/acsami.5b01447 25966790

[14] Matos AC, Marques CF, Pinto RV, Ribeiro IAC, Gonçalves LM, Vaz MA, et al. Novel doped calcium phosphate-PMMA bone cement composites as levofloxacin delivery systems. Int J Pharm. 2015;490(1–2):200–8. doi: 10.1016/j.ijpharm.2015.05.038 26002570

[15] Díez-Pascual AM. PMMA-Based nanocomposites for odontology applications: a state-of-the-art. Int J Mol Sci. 2022;23(18):10288. doi: 10.3390/ijms231810288 36142201 PMC9499310

[16] Xu D, Esko JD. Demystifying heparan sulfate-protein interactions. Annu Rev Biochem. 2014;83:129–57. doi: 10.1146/annurev-biochem-060713-035314 24606135 PMC7851832

[17] Hettiaratchi MH, Rouse T, Chou C, Krishnan L, Stevens HY, Li M-TA, et al. Enhanced in vivo retention of low dose BMP-2 via heparin microparticle delivery does not accelerate bone healing in a critically sized femoral defect. Acta Biomater. 2017;59:21–32. doi: 10.1016/j.actbio.2017.06.028 28645809 PMC6546418

[18] Weiss RJ, Esko JD, Tor Y. Targeting heparin and heparan sulfate protein interactions. Org Biomol Chem. 2017;15(27):5656–68. doi: 10.1039/c7ob01058c 28653068 PMC5567684

[19] Esko JD, Lindahl U. Molecular diversity of heparan sulfate. J Clin Invest. 2001;108:169–73. doi: 10.1172/jci200113530 11457867 PMC203033

[20] Liu J, Linhardt RJ. Chemoenzymatic synthesis of heparan sulfate and heparin. Nat Prod Rep. 2014;31(12):1676–85. doi: 10.1039/c4np00076e 25197032 PMC4387879

[21] Kratz G, Back M, Arnander C, Larm O. Immobilised heparin accelerates the healing of human wounds in vivo. Scand J Plast Reconstr Surg Hand Surg. 1998;32(4):381–5. doi: 10.1080/02844319850158462 9862105

[22] Kashiwakura Y, Kojima H, Kanno Y, Hashiguchi M, Kobata T. Heparin affects the induction of regulatory T cells independent of anti-coagulant activity and suppresses allogeneic immune responses. Clin Exp Immunol. 2020;202(1):119–35. doi: 10.1111/cei.13480 32562271 PMC7488123

[23] Rasmark Roepke E, Bruno V, Nedstrand E, Boij R, Strid CP, Piccione E, et al. Low-molecular-weight-heparin increases Th1- and Th17-associated chemokine levels during pregnancy in women with unexplained recurrent pregnancy loss: a randomised controlled trial. Sci Rep. 2019;9(1):12314. doi: 10.1038/s41598-019-48799-6 31444404 PMC6707182

[24] Palmqvist P, Lundberg P, Persson E, Johansson A, Lundgren I, Lie A, et al. Inhibition of hormone and cytokine-stimulated osteoclastogenesis and bone resorption by interleukin-4 and interleukin-13 is associated with increased osteoprotegerin and decreased RANKL and RANK in a STAT6-dependent pathway. J Biol Chem. 2006;281(5):2414–29. doi: 10.1074/jbc.M510160200 16251181

[25] Papy-Garcia D, Albanese P. Heparan sulfate proteoglycans as key regulators of the mesenchymal niche of hematopoietic stem cells. Glycoconj J. 2017;34(3):377–91. doi: 10.1007/s10719-017-9773-8 28577070

[26] Xu Z, Chen S, Feng D, Liu Y, Wang Q, Gao T, et al. Biological role of heparan sulfate in osteogenesis: a review. Carbohydr Polym. 2021;272:118490. doi: 10.1016/j.carbpol.2021.118490 34420746

[27] Hu J, Wang Z, Miszuk JM, Zeng E, Sun H. High molecular weight poly(glutamic acid) to improve BMP2-induced osteogenic differentiation. Molecular Pharm. 2022;19(12):4565–75. doi: 10.1021/acs.molpharmaceut.2c00141 35675584 PMC9729371

[28] Jackson RA, Murali S, van Wijnen AJ, Stein GS, Nurcombe V, Cool SM. Heparan sulfate regulates the anabolic activity of MC3T3-E1 preosteoblast cells by induction of Runx2. J Cell Physiol. 2007;210(1):38–50. doi: 10.1002/jcp.20813 17051597

[29] Ling L, Murali S, Stein GS, van Wijnen AJ, Cool SM. Glycosaminoglycans modulate RANKL-induced osteoclastogenesis. J Cell Biochem. 2010;109(6):1222–31. doi: 10.1002/jcb.22506 20135643 PMC3095103

[30] Liu Y, Wang R, Chen S, Xu Z, Wang Q, Yuan P, et al. Heparan sulfate loaded polycaprolactone-hydroxyapatite scaffolds with 3D printing for bone defect repair. Int J Biol Macromol. 2020;148:153–62. doi: 10.1016/j.ijbiomac.2020.01.109 31935409

[31] Ding L, Hao K, Sang L, Shen X, Zhang C, Fu D, et al. ATF2-driven osteogenic activity of enoxaparin sodium-loaded polymethylmethacrylate bone cement in femoral defect regeneration. J Orthop Surg Res. 2023;18(1):646. doi: 10.1186/s13018-023-04017-8 37653390 PMC10470168

[32] Hao K, Sang L, Ding L, Shen X, Fu D, Qi X. Enoxaparin sodium bone cement displays local anti-inflammatory effects by regulating the expression of IL-6 and TNF-α. Heliyon. 2023;9(6):e16530. doi: 10.1016/j.heliyon.2023.e16530 37274684 PMC10238720

[33] Casele H, Haney EI, James A, Rosene-Montella K, Carson M. Bone density changes in women who receive thromboprophylaxis in pregnancy. Am J Obstet Gynecol. 2006;195(4):1109–13. doi: 10.1016/j.ajog.2006.06.080 17000242

[34] Xiao Z, Fu D, Zhang L, Fan W, Shen X, Qi X. Bone healing study of alendronate combined with enoxaparin sodium bone cement in rabbits with bone defects. J Orthop Surg Res. 2022;17(1):431. doi: 10.1186/s13018-022-03330-y 36175933 PMC9524070

[35] Sun H, Ma X, Li Z, Liu J, Wang W, Qi X. Release characteristics of enoxaparin sodium-loaded polymethylmethacrylate bone cement. J Orthop Surg Res. 2021;16(1):108. doi: 10.1186/s13018-021-02223-w 33541384 PMC7860616

[36] Fan W, Fu D, Zhang L, Xiao Z, Shen X, Chen J, et al. Enoxaparin sodium bone cement plays an anti-inflammatory immunomodulatory role by inducing the polarization of M2 macrophages. J Orthop Surg Res. 2023;18(1):380. doi: 10.1186/s13018-023-03865-8 37221568 PMC10207791

[37] Sang L, Hao K, Ding L, Shen X, Sun H, Fu D, et al. The mechanism by which enoxaparin sodium-high-viscosity bone cement reduces thrombosis by regulating CD40 expression in endothelial cells. BMC Musculoskelet Disord. 2022;23(1):513. doi: 10.1186/s12891-022-05469-5 35637498 PMC9150327

[38] Aziz SZ, Jafar ZJ. The efficacy of little lovely dentist and tell show do in alleviating dental anxiety in Iraqi children: a randomized clinical trial. J Int Soc Prev Community Dent. 2023;13(5):388–93. doi: 10.4103/jispcd.JISPCD_112_23 38124727 PMC10729888

[39] Wang Y, Zhang H, Hu Y, Jing Y, Geng Z, Su J. Bone repair biomaterials: A perspective from immunomodulatory. Adv Funct Mater. 2022;2022:2208639.

[40] Chen Q, Zhang X, Zhang D, Liu G, Ma K, Liu J, et al. Universal and one-step modification to render diverse materials bioactivation. J Am Chem Soc. 2023;145(32):18084–93. doi: 10.1021/jacs.3c05928 37527432

[41] Ji X, Xu F, Dong G, Jia C, Jia P, Chen H, et al. Loading necrostatin-1 composite bone cement inhibits necroptosis of bone tissue in rabbit. Regen Biomater. 2019;6(2):113–9. doi: 10.1093/rb/rbz004 30967966 PMC6447002

[42] Medeiros CCG, Borghetti RL, Nicoletti N, da Silva VD, Cherubini K, Salum FG, et al. Polymethylmethacrylate dermal fillers: evaluation of the systemic toxicity in rats. Int J Oral Maxillofac Surg. 2014;43(1):62–7. doi: 10.1016/j.ijom.2013.06.009 23871301

[43] Tatsumi S, Ishii K, Amizuka N, Li M, Kobayashi T, Kohno K, et al. Targeted ablation of osteocytes induces osteoporosis with defective mechanotransduction. Cell Metab. 2007;5(6):464–75. doi: 10.1016/j.cmet.2007.05.001 17550781

[44] Tomecka MJ, Ethiraj LP, Sánchez LM, Roehl HH, Carney TJ. Clinical pathologies of bone fracture modelled in zebrafish. Dis Model Mech. 2019;12(9):dmm037630. doi: 10.1242/dmm.037630 31383797 PMC6765199

[45] Prystaz K, Kaiser K, Kovtun A, Haffner-Luntzer M, Fischer V, Rapp AE, et al. Distinct effects of IL-6 classic and trans-signaling in bone fracture healing. Am J Pathol. 2018;188(2):474–90. doi: 10.1016/j.ajpath.2017.10.011 29146294

[46] Guo W, Yan S, Zhao G. Upregulated ATF1 promotes lipopolysaccharide induced inflammatory response and inhibits osteogenic differentiation of human periodontal ligament cells by regulating NF-κB pathway. Discov Med. 2024;36(182):518–26. doi: 10.24976/Discov.Med.202436182.48 38531792

[47] Lee J, Lee E, Huh SJ, Kang JI, Park KM, Byun H, et al. Composite spheroid-laden bilayer hydrogel for engineering three-dimensional osteochondral tissue. Tissue Eng Part A. 2024;30(5–6):225–43. doi: 10.1089/ten.TEA.2023.0299 38062771

[48] Xiong Q, Zhang L, Ge W, Tang P. The roles of interferons in osteoclasts and osteoclastogenesis. Joint Bone Spine. 2016;83(3):276–81. doi: 10.1016/j.jbspin.2015.07.010 26832190

[49] Zheng D, Liwinski T, Elinav E. Inflammasome activation and regulation: toward a better understanding of complex mechanisms. Cell Discov. 2020;6:36. doi: 10.1038/s41421-020-0167-x 32550001 PMC7280307

[50] Wang R, Li H, Wu J, Cai Z-Y, Li B, Ni H, et al. Gut stem cell necroptosis by genome instability triggers bowel inflammation. Nature. 2020;580(7803):386–90. doi: 10.1038/s41586-020-2127-x 32296174

[51] Zhou T, Cao J, Tang Q, Jin J, Liang Y, Feng B. Exploring the role of NAA40 in immune infiltrates and prognostic prediction in hepatocellular carcinoma. Am J Clin Exp Immunol. 2024;13(1):26–34. 38496356 10.62347/UGPH7404 PMC10944357

[52] Zhou H-Y, Wang Y-C, Wang T, Wu W, Cao Y-Y, Zhang B-C, et al. CCNA2 and NEK2 regulate glioblastoma progression by targeting the cell cycle. Oncol Lett. 2024;27(5):206. doi: 10.3892/ol.2024.14339 38516683 PMC10956385

[53] Yu C-L, Lu F, Yu D-H, Xu X-M, Xu P, Liu S-M. Mechanism of acteoside in prevention and treatment of gouty arthritis based on liver metabolomics. Zhongguo Zhong Yao Za Zhi. 2024;49(1):224–31. doi: 10.19540/j.cnki.cjcmm.20230808.402 38403355

[54] Chovatia RM, Acharya A, Rasal KD, Bedekar MK, Jeena K, Rathinam RB, et al. Ontogeny and tissue specific expression profiles of recombination activating genes (RAGs) during development in Nile tilapia, Oreochromis niloticus. Gene Expr Patterns. 2024;52:11. doi: 10.1016/j.gep.2024.119358 38460579

[55] Villa A, Santagata S, Bozzi F, Giliani S, Frattini A, Imberti L, et al. Partial V(D)J recombination activity leads to Omenn syndrome. Cell. 1998;93(5):885–96. doi: 10.1016/s0092-8674(00)81448-8 9630231

[56] Santagata S, Gomez CA, Sobacchi C, Bozzi F, Abinun M, Pasic S, et al. N-terminal RAG1 frameshift mutations in Omenn’s syndrome: internal methionine usage leads to partial V(D)J recombination activity and reveals a fundamental role in vivo for the N-terminal domains. Proc Natl Acad Sci U S A. 2000;97(26):14572–7. doi: 10.1073/pnas.97.26.14572 11121059 PMC18960

[57] Noordzij JG, de Bruin-Versteeg S, Verkaik NS, Vossen JMJJ, de Groot R, Bernatowska E, et al. The immunophenotypic and immunogenotypic B-cell differentiation arrest in bone marrow of RAG-deficient SCID patients corresponds to residual recombination activities of mutated RAG proteins. Blood. 2002;100(6):2145–52. 12200379

[58] Wu Z, You C, Zhu Z, Wu W, Cao J, Xie Q, et al. SLA2 is a prognostic marker in HNSCC and correlates with immune cell infiltration in the tumor microenvironment. Eur Arch Otorhinolaryngol. 2024;281(1):427–40. doi: 10.1007/s00405-023-08213-4 37688682 PMC10764518

[59] Dragone LL, Shaw LA, Myers MD, Weiss A. SLAP, a regulator of immunoreceptor ubiquitination, signaling, and trafficking. Immunol Rev. 2009;232(1):218–28. doi: 10.1111/j.1600-065X.2009.00827.x 19909366

[60] Wybenga-Groot LE, McGlade CJ. RTK SLAP down: the emerging role of Src-like adaptor protein as a key player in receptor tyrosine kinase signaling. Cell Signal. 2015;27(2):267–74. doi: 10.1016/j.cellsig.2014.11.010 25446260

[61] Myers MD, Sosinowski T, Dragone LL, White C, Band H, Gu H, et al. Src-like adaptor protein regulates TCR expression on thymocytes by linking the ubiquitin ligase c-Cbl to the TCR complex. Nat Immunol. 2006;7(1):57–66. doi: 10.1038/ni1291 16327786

[62] Holland SJ, Liao XC, Mendenhall MK, Zhou X, Pardo J, Chu P, et al. Functional cloning of Src-like adapter protein-2 (SLAP-2), a novel inhibitor of antigen receptor signaling. J Exp Med. 2001;194(9):1263–76. doi: 10.1084/jem.194.9.1263 11696592 PMC2195979

[63] Grzybowska EA. Calcium-binding proteins with disordered structure and their role in secretion, storage, and cellular signaling. Biomolecules. 2018;8(2):42. doi: 10.3390/biom8020042 29921816 PMC6022996

[64] Michetti F, Di Sante G, Clementi ME, Sampaolese B, Casalbore P, Volonté C, et al. Growing role of S100B protein as a putative therapeutic target for neurological- and nonneurological-disorders. Neurosci Biobehav Rev. 2021;127:446–58. doi: 10.1016/j.neubiorev.2021.04.035 33971224

[65] Michetti F, Clementi ME, Di Liddo R, Valeriani F, Ria F, Rende M, et al. The S100B protein: a multifaceted pathogenic factor more than a biomarker. Int J Mol Sci. 2023;24(11):9605. doi: 10.3390/ijms24119605 37298554 PMC10253509

[66] Bertheloot D, Latz E. HMGB1, IL-1α, IL-33 and S100 proteins: dual-function alarmins. Cell Mol Immunol. 2017;14(1):43–64. doi: 10.1038/cmi.2016.34 27569562 PMC5214941

[67] Lember L-M, Ntikas M, Mondello S, Wilson L, Di Virgilio TG, Hunter AM, et al. The use of biofluid markers to evaluate the consequences of sport-related subconcussive head impact exposure: a scoping review. Sports Med Open. 2024;10(1):12. doi: 10.1186/s40798-023-00665-6 38270708 PMC10811313

[68] Kleindienst A, Tolias CM, Corwin FD, Müller C, Marmarou A, Fatouros P, et al. Assessment of cerebral S100B levels by proton magnetic resonance spectroscopy after lateral fluid-percussion injury in the rat. J Neurosurg. 2005;102(6):1115–21. doi: 10.3171/jns.2005.102.6.1115 16028772

