# Supplementary material for: Mechanisms responsible for the ability of enoxaparin sodium to inhibit inflammatory responses in the immune microenvironment of bone repair: A transcriptomic sequencing study
Source: PLoS One. 2025 Sep 8;20(9):e0332041. doi: 10.1371/journal.pone.0332041 (PMC12416700; doi:10.1371/journal.pone.0332041)
Supplement: S1 File — Supplementary Figure 1. Precision-Recall and ROC Curves for LASSO, SVM, and Boruta Models. (A, B) (LASSO Model): Accuracy = 1.0 (100%) | Recall = 1.0 (100%) | AUC-ROC = 1.0 (Perfect classification). (C, D) (SVM Model): Accuracy = 0.909 (90.9%) | Recall = 1.0 (100%) | AUC-ROC = 1.0 (Perfect classification). (E, F) (Boruta Model): Accuracy = 1.0 (100%) | Recall = 1.0 (100%) | AUC-ROC = 1.0 (Perfect classification). Supplementary Figure 2. ES-PMMA exhibits anti-inflammatory effects by modulating S100B, SLA2 and RAG1 expression in validation experiments. (A) S100B expression was significantly lower in ES-PMMA compared to PMMA (*p < 0.0001) and Model (p = 0.0016). (B) SLA2 expression showed suppression in Model (*p = 0.0008) and PMMA (p = 0.0001) versus Control, with partial reversal by ES-PMMA (*p = 0.008 vs PMMA). (C) RAG1 expression was reduced in ES-PMMA versus PMMA (*p = 0.035) and Model (p = 0.0098). Data shown as mean ± SEM (n = 6/group). One-way ANOVA with Tukey’s multiple comparisons test was used for all comparisons. *p < 0.05, **p < 0.01, ***p < 0.001. (DOCX) [file pone.0332041.s001.docx]

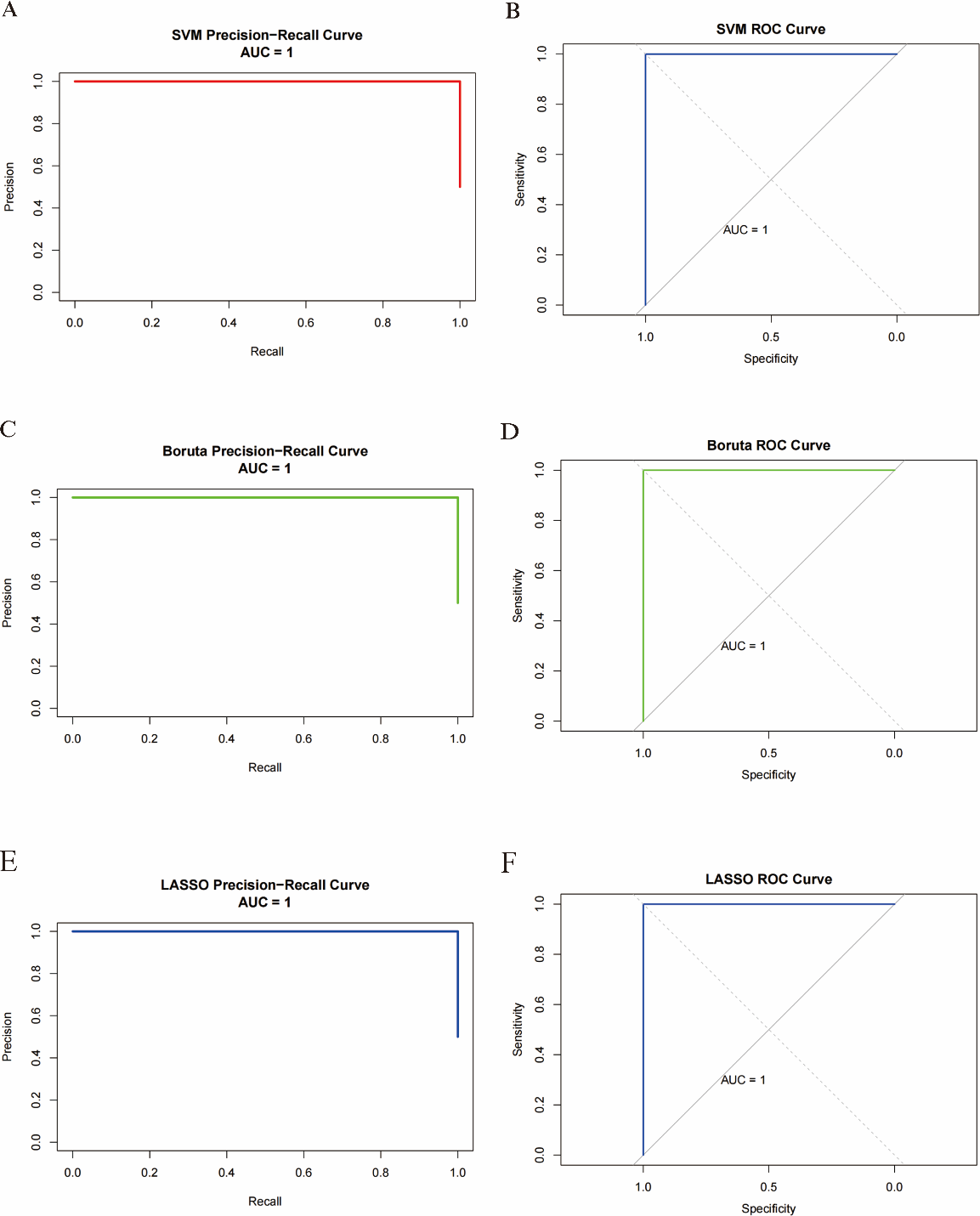


**Supplementary Figure 1.** Precision-Recall and ROC Curves for LASSO, SVM, and Boruta Models. (A, B) (LASSO Model): Accuracy = 1.0 (100%) | Recall = 1.0 (100%) | AUC-ROC = 1.0 (Perfect classification). (C, D) (SVM Model): Accuracy = 0.909 (90.9%) | Recall = 1.0 (100%) | AUC-ROC = 1.0 (Perfect classification). (E, F) (Boruta Model): Accuracy = 1.0 (100%) | Recall = 1.0 (100%) | AUC-ROC = 1.0 (Perfect classification).


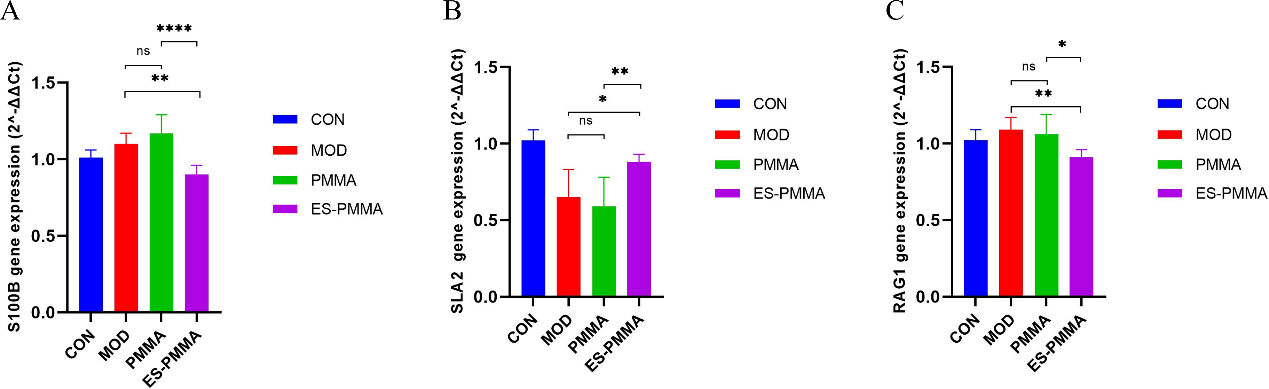


**Supplementary Figure 2.** ES-PMMA exhibits anti-inflammatory effects by modulating S100B, SLA2 and RAG1 expression in validation experiments. (A) S100β expression was significantly lower in ES-PMMA compared to PMMA (*p < 0.0001) and Model (p = 0.0016). (B) SLA2 expression showed suppression in Model (*p = 0.0008) and PMMA (p = 0.0001) versus Control, with partial reversal by ES-PMMA (*p = 0.008 vs PMMA). (C) RAG1 expression was reduced in ES-PMMA versus PMMA (*p = 0.035) and Model (p = 0.0098). Data shown as mean ± SEM (n=6/group). One-way ANOVA with Tukey's multiple comparisons test was used for all comparisons. *p<0.05, **p<0.01, ***p<0.001.
